# A systematic review of the effect of therapeutic drug monitoring on patient health outcomes during treatment with penicillins

**DOI:** 10.1093/jac/dkac101

**Published:** 2022-03-31

**Authors:** Timothy Luxton, Natalie King, Christoph Wälti, Lars Jeuken, Jonathan Sandoe

**Affiliations:** 1 School of Biomedical Sciences, University of Leeds, Leeds LS2 9JT, UK; 2 Leeds Institute of Health Sciences, University of Leeds, Leeds LS2 9JT, UK; 3 School of Electronic and Electrical Engineering, University of Leeds, Leeds LS2 9JT, UK; 4 Leiden Institute of Chemistry, Leiden University, PO Box 9502, 2300 RA, Leiden, The Netherlands; 5 School of Medicine, University of Leeds, Leeds LS2 9JT, UK

## Abstract

**Background:**

Dosing regimens guided by therapeutic drug monitoring (TDM) may be able to improve penicillin exposure in patients, which could result in improved patient health outcomes.

**Objectives:**

This systematic review aims to describe the impact penicillin TDM has on health outcomes, including antimicrobial resistance (AMR).

**Methods:**

Studies measuring penicillins in patient samples that adjusted regimens according to the result, and reported health outcomes were selected. Study bias was assessed according to study type. Included study characteristics were tabulated and described by narrative synthesis.

**Results:**

Three randomized controlled trials (RCTs), 16 cohort studies, and 9 case studies were included. No RCTs showed statistically significant improvements in health outcomes. Five cohort studies showed improvement in at least one health outcome associated with target attainment. However, there was a high risk of bias in all studies for health outcomes. One study assessed the impact of penicillin TDM on AMR and found that improved target attainment was associated with suppression of resistance. No studies found a detrimental effect of penicillin TDM.

**Conclusions:**

There is little evidence to suggest that TDM improves health outcomes, however neither health outcomes nor impact on AMR were adequately addressed. Variations in TDM implementation meant that a meta-analysis was not suitable. Penicillin TDM needs standardization, however there is currently no clear evidence of optimal conditions. Suitably powered studies are required to resolve the ambiguity surrounding the impact of TDM on clinical outcomes, including AMR. Further, standardized protocols and concentration targets need to be identified for TDM to be implemented successfully.

## Introduction

Antibiotics are crucially important for the treatment of bacterial infection.^1^ Penicillins are an important class of antibiotics, with agents such as amoxicillin, ampicillin and piperacillin being the most commonly consumed agents globally.^2^ The consumption of these penicillins is increasing globally, with a 36% increase in defined daily doses observed between 2000 and 2015.^2^ Antibiotic regimens are commonly prescribed empirically, depending on the clinical diagnosis and the most likely causative pathogens.^3^ Dosing regimens are usually standardized, based on data from studies that test pharmacokinetics and pharmacodynamics (PK/PD) in healthy volunteers.^3^ However, a substantial body of research shows there is significant pharmacological variability of β-lactam antibiotics, including penicillins, between patients; particularly in critically ill patients.^4–8^ Such pharmacological variability may result in unsuitable dosing regimens (both under-dosing and over-dosing) potentially leading to less effective treatment,^9^ toxicity,^10^ and emergence of antimicrobial resistance (AMR).^11^

Therapeutic drug monitoring (TDM) is the practice of measuring a chemical parameter, a drug or a biomarker, and altering the dosing regimen based on the result.^12^ While there is evidence that penicillin TDM can improve pharmacological target attainment, with potential to improve patient outcomes, TDM is not currently widely used during treatment of patients with penicillins.^4^ Penicillins exert their antibiotic action in a time-dependent manner; the longer the concentration is above the MIC, the greater the bactericidal effect.^13^ The MIC is the concentration threshold of an antibiotic sufficient to prevent bacterial growth and it is MIC data that are used to determine therapeutic targets.^13^ Further, a fraction of the administered antibiotic will be reversibly bound by blood proteins and a fraction will remain free, known as the unbound fraction. Therapeutic targets are commonly based around the amount of time unbound antibiotic remains above the MIC, denoted as *fT*_>MIC_.^14^*fT*_>MIC_ and other PK/PD indices such as *C*_min/max_/MIC, the ratio between the trough/peak concentration and the MIC, can be used in TDM to optimize antibiotic effect.^14^ TDM of penicillins, however, is a complex intervention, with a number of interacting components.^15^ Firstly, taking the sample; how soon into treatment the first sample is taken, the time of sampling compared with the previous dose, the frequency of sampling, whether steady-state PK/PD is reached, and sampling area. Distribution of penicillins differs in different parts of the body, the most appropriate sampling site will most likely depend on the indication of infection. Secondly, sample processing, measurement of the sample, and reporting the result; the stability of the penicillin throughout sample processing and quantification; the length of time it takes from taking the sample to reporting a result and the relevance of the result after that time; and the accuracy of quantification. Thirdly, what is done with the result to change treatment; the effectiveness of dose adjustment protocols to alter the antibiotic concentration sufficiently in the area of infection; the concentration target range and whether that is based on the MIC of the individual infection, or on worst-case scenario species-specific or non-species-specific clinical breakpoints; whether dose adjustment recommendations are followed by clinicians; and subsequent antibiotic measurements to ensure that the antibiotic concentrations remains in the target range.

Keeping concentrations in target ranges may have impacts on antimicrobial resistance, which is a global concern. It is predicted that there will be 10 million global deaths per year due to antimicrobial resistant infections by 2050.^16^ Microbes evolve resistance to antimicrobials because the use of antimicrobials introduces a selection pressure where, through mutation and gene transfer, microbes develop mechanisms to survive in the presence of antimicrobial agents.^17^ Since unsuitable dosing regimens may exacerbate AMR, ensuring optimized dosing regimens through use of TDM may reduce emergence of AMR. This systematic review aimed to summarize the current published literature on the effect of penicillin TDM on clinical outcomes and the emergence of AMR, and to answer whether or not penicillin TDM improves health outcomes.

## Methods

The reporting of this review has followed the Preferred Reporting Items for Systematic Reviews and Meta-Analyses (PRISMA) 2020 guidelines.^18^ The protocol was registered on PROSPERO (ID: CRD42020202800).

The MEDLINE, EMBASE, Cochrane, and Web of Science databases were initially searched from inception to 26 June 2020 to identify studies investigating penicillins and TDM. Following study selection and data extraction of the initial search, the search was repeated for studies published between the start of 2020 and 27 September 2021. Carbapenems were also included in the search strategy but were analysed separately and will be presented in a separate systematic review. The search terms and strategy are shown in Supplementary data (available at *JAC* Online). Following the database search, a hand search of the reference lists of relevant reviews was conducted.^4,19–30^

### Definitions

Based on a previous definition, TDM was defined as the measurement of an antibiotic that was used by healthcare professionals to alter the administration of the drug (dose, frequency or route).^31^

### Inclusion and exclusion criteria

Population: Patients treated with penicillin antibiotics. Condition: Suspected or confirmed infection, as indicated by treatment with penicillins. Intervention: Studies that used TDM to modify the dosing regimen of the prescribed penicillin. Comparator/control: Patients treated with standard care, where dosing regimens were not influenced by TDM results. Type of study: randomized controlled trials (RCTs), non-randomized trials (cohort studies and quasi-experimental studies), retrospective and observational studies were included. Exclusion criteria were: Studies not written in the English language; not related to penicillins; studies not addressing clinical outcomes; animal studies; conference abstracts; reviews.

### Selection and data extraction

EndNote X9 software (Clarivate Analytics) was used to deduplicate and manage all references obtained from the searches. Reference title and abstracts were screened by a single reviewer (T.L.). Using a random number generator, 10% of the references were randomly selected and the title and abstracts were screened by a second reviewer (J.S.). Any discrepancies between the selections were resolved through discussion with all authors. Following the title and abstract screen, a full-text screen was performed by reviewer T.L. with 10% randomly selected for screening by the second reviewer (J.S.).

### Data collection

Data was collected by hand by reviewer T.L., data collection was then validated by the second reviewer (J.S.). Collected intervention data consisted of: antibiotic, administration method, dose and dose frequency, duration of therapy, antibiotic quantification method, bodily fluid used for antibiotic quantification (e.g. saliva, plasma, interstitial fluid), the fraction quantified (the free or total fraction), frequency of quantification, dose adjustment protocol, planned antibiotic target level, and target attainment. Study data collected consisted of: study type, population, population size, intervention group size, control group size, microbiological confirmation of infection, pathogen(s), study location (in hospital or community based study). Outcome data collected consisted of: mortality, in-hospital stay, length of stay on ICU, acute kidney injury, toxicity or other adverse effects, treatment efficacy (that is the resolution of signs or symptoms of infection), duration of treatment, readmission, target attainment, readmission, emergence of antibiotic resistance. If there were any outcomes not reported, or missing study characteristics, it was assumed that they were not collected or assessed. Studies with missing summary data or missing outcome data were assessed in the quality assessment.

### Quality assessment

Risk of bias analysis was carried out independently by two reviewers (T.L. and J.S.) using the following tools: for RCTs the Revised Cochrane Risk-of-Bias Tool for Randomised Trials (RoB2) assessment tool was used. For non-randomized intervention studies the Risk of Bias in Non-Randomised Studies (ROBINS-1) assessment tool was used. For case studies the Office of Health Assessment and Translation (OHAT) assessment tool was used.

### Data analysis

Studies were assigned to one of three categories based on the level of evidence, RCTs, non-randomized cohort studies, and case studies. Studies were grouped by study design to minimize impact of confounding factors on RCT results, as per guidance.^32^ The study characteristics were tabulated, and a narrative synthesis was performed. Study characteristics such as: pharmacological targets, dose adjustment protocols, sampling, co-administered antibiotics, susceptibility definitions were compared to assess study synthesis suitability. Health outcomes of studies that included a comparison group, RCTs and non-randomized studies, were tabulated. For studies with a low risk of bias, a meta-analysis would be carried out where pharmacological targets and dose adjustment protocols were consistent.

## Results

### Search results

**Figure 1.**
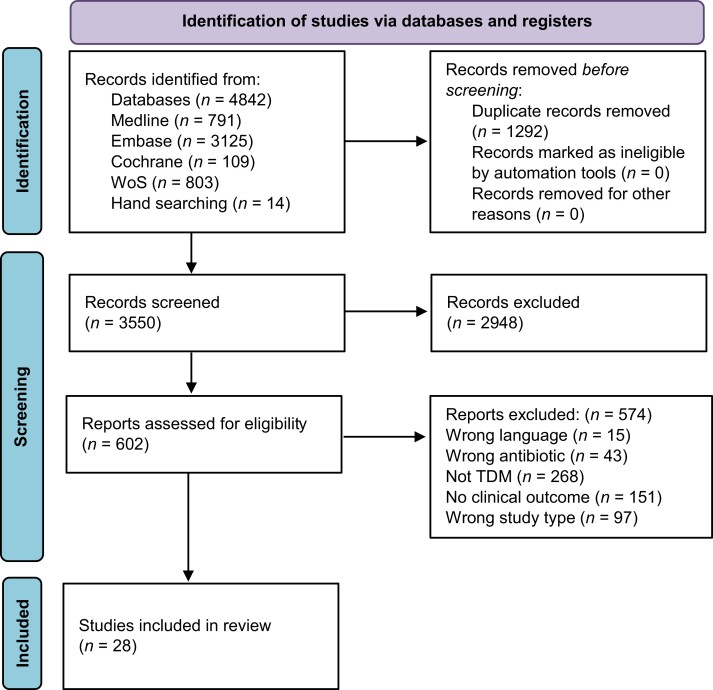
Literature search process in accordance with the Preferred Reporting Items for Systematic Reviews and Meta-Analyses (PRISMA) guidelines.^18^ This figure appears in colour in the online version of *JAC* and in black and white in the print version of *JAC*.

Figure 1 outlines the results of the search strategy. The database searches identified 4842 studies. Once duplicates were removed there were 3550 records. Hand searching identified 14 records. The screen by title and abstract identified 602 records for full text review and 28 studies were eligible for inclusion.

### Included studies

Twenty-eight studies fulfilled the inclusion criteria. Of these, 3 were RCTs, 16 were non-randomized cohort studies, and 9 were case studies. Some studies that measured penicillin concentrations in patients and reported clinical outcomes appeared to meet the inclusion criteria, but were excluded as they did not adjust patient dosing regimens according to penicillin quantification result, as specified by the TDM definition used.^31^

### Randomized controlled trials

#### Quality assessment

Results of the risk of bias assessment using the RoB2 assessment tool are shown in Table 1. Each study scored ‘Some Concerns’ or ‘High’ levels of bias. Bias in the randomization process occurred due to not specifying randomization procedures or allocation concealment processes.^33,34^ Bias in deviations from intended interventions was seen where co-interventions such as concomitant antibiotics were not specified^33,34^ and where the effects of deviations from the intended treatment were not assessed appropriately.^34^ The studies all scored ‘Some Concerns’ in the ‘bias in selection of reported result’ section, since there were no available protocols of the studies before the published RCTs.^33–35^

**Table 1.** Revised Cochrane Risk-of-Bias tool for randomized controlled trials (RoB2)

Study	Bias in randomization process	Bias in deviations from intended interventions (effect of assignment to intervention)	Bias in deviations from intended interventions (effect of adhering to intervention)	Bias due to missing outcome data	Bias due to measurement of outcome	Bias in selection of reported result	Overall Result
Sime *et al.* 2015.^35^	Low	Low	Low	Low	Low	Some Concerns	Some Concerns
Fournier *et al.* 2018.^34^	Some Concerns	Low	High	Low	High	Some Concerns	High
De Waele *et al.* 2014.^33^	Some Concerns	Low	High	Low	Low	Some Concerns	High

#### Study characteristics and results

Table S1 (see Supplementary data at *JAC* Online) summarizes the characteristics of the eligible RCTs, which included febrile neutropenia patients,^35^ burns patients,^34^ and patients with normal kidney function.^33^ These studies showed little difference in clinical outcomes between standard care patients, and those who received TDM-guided treatment.

Sime *et al.*^35^ carried out an RCT that investigated TDM use in 32 febrile neutropenic patients with haematological malignancies who were administered piperacillin/tazobactam. TDM was implemented in the treatment of 16 patients whilst 16 patients received standard care.^35^ The study showed that TDM-guided piperacillin administration resulted in improved pharmacological target attainment from the second day of treatment: a higher proportion of TDM measurements fell within the PK/PD target (100% *fT*_>MIC_) in the intervention group compared with the control, first TDM *P *= 1.00, second TDM *P *= 0.012, and third TDM *P *= 0.004.^35^ Despite the improvement in PK/PD target attainment there was no difference in the clinical outcomes between the two groups. In both groups the median duration of fever was 2 days (control IQR: 1–4 days, intervention IQR: 1–3 days) and recovery time from neutropenia was 6 days (control IQR: 4–13 days, intervention IQR: 3–8 days).^35^

Fournier *et al.*^34^ performed an RCT that looked at the impact of TDM of several β-lactams in 38 burns patients, 30 of whom were administered penicillins. There was no significant improvement in target attainment between the TDM intervention group and the standard care group in relation to penicillins. The study showed no difference in clinical outcomes. Infection resolution in the intervention group was 91.7% and infection resolution in the standard care group was 96.8%; however, this included infections treated with other β-lactams and it was not possible to separate out the impact on therapy with penicillins.

The RCT conducted by De Waele *et al.*^33^ investigated the difference TDM made to target attainment in 41 patients treated with piperacillin/tazobactam (*n *= 28) and meropenem (*n *= 13), with normal renal function. Target attainment was analysed from antibiotic measurements at baseline and again after 3 days. Target attainment in the intervention group improved significantly compared with the standard care group. Despite the improvement in target attainment overall, there was no significant difference in clinical outcomes seen from TDM use. Clinical failure was seen in two patients in the intervention group and two in the control group (*P *= 0.41). Persistence of the bacterial pathogen at day 7 was seen in one patient in the intervention group and five in the control group (*P *= 0.09), suggesting a trend towards improved microbiological outcome. Median SOFA scores in the intervention group changed from 5.5 at the baseline to 3 at day 7 (*P *= 0.093). In the control group the median SOFA score change was from 5 to 4, from baseline to day 7 (*P *= 0.575). In patients treated with piperacillin, 4.8% (*n *= 1) of patients in the intervention group died in the ICU compared with 20% (*n *= 4) in the control group (*P *= 0.18). There was no significant difference in 28 day mortality in piperacillin patients, where two patients died in the intervention group compared with four deaths in the control group. PK data and clinical data were reported for the whole intervention group (i.e. patients treated with meropenem and piperacillin/tazobactam), mortality data for piperacillin and meropenem were provided by the author.

### Non-randomized studies

#### Quality assessment

Results of the risk of bias assessment are shown in Table 2. All of these studies scored a serious or critical risk of bias in the context of this research question. The majority of these studies (69%) did not perform any analysis of the effects of confounding factors. Almost all of the studies had a low risk of bias in patient selection. Since the majority of these studies were observational studies, where all the included patients received the same intervention, bias due to classification of intervention was not applicable.

**Table 2.** Quality assessment of non-randomized studies as per the ROBINS-I assessment tool^54^

Study	Bias due to confounding	Bias in selection of participants into the study	Bias in classification of interventions	Bias due to deviations from intended interventions	Bias due to missing data	Bias in measurement of outcomes	Bias in selection of the reported result	Overall result
Cies *et al.* 2018.^39^	Critical	Low	NA	Serious	Low	Low	Moderate	Critical
Duszynska, 2012.^51^	Critical	Low	NA	Critical	Low	Low	Moderate	Critical
Economou *et al.* 2017.^46^	Critical	Low	NA	Serious	Low	Low	Moderate	Critical
Besnard *et al.* 2019.^47^	Critical	Low	NA	Serious	Low	Low	Moderate	Critical
McDonald *et al.* 2016.^37^	Critical	Critical	NA	NI	Low	Serious	Moderate	Critical
Wong *et al.* 2018.^36^	Critical	Low	NA	Critical	Moderate	Serious	Moderate	Critical
Patel *et al.* 2012.^38^	Critical	Low	NA	NI	Low	Serious	Moderate	Critical
Roberts *et al.* 2010.^42^	Critical	Low	NA	Serious	Low	Low	Moderate	Critical
Richter *et al.* 2019.^40^	Critical	Low	NA	Serious	Low	Low	Serious	Critical
Machado *et al.* 2017.^50^	Serious	Low	Low	NI	Low	Low	Moderate	Serious
Schoenenberger-Arnaiz *et al.* 2019.^41^	Critical	Low	NA	Critical	Low	Low	Moderate	Critical
Jansen *et al.* 2021.^48^	Critical	Low	NA	Critical	Low	Low	Moderate	Critical
Gomez-Junyent *et al.* 2020.^49^	Critical	Low	NA	Serious	Low	Low	Moderate	Critical
Chiriac *et al.* 2021.^43^	Serious	Low	NA	Moderate	Low	Low	Moderate	Serious
Al-Shaer *et al.* 2020.^44^	Serious	Critical	NA	Critical	Serious	Low	Moderate	Critical
Scharf *et al.* 2020.^45^	Moderate	Critical	NA	Critical	Serious	Low	Moderate	Critical

NI, not enough information reported; NA, not applicable.

A key area of bias in all the studies was deviation from the intended intervention. High risk of bias was seen due to unassessed impacts of co-interventions, such as concomitant antibiotics and renal replacement therapy (RRT), and unsuccessful interventions, where it was shown that dose adjustments guided by TDM result were ineffective. Bias due to missing data was generally low. There was minimal risk of bias due to measuring outcomes since the outcomes measured were usually objective. However, there was a serious risk of bias scored when definitions for clinical outcome, or clinical improvement leading to escalation/de-escalation of treatment were considered subjective.^36–38^ Most studies scored a moderate risk of bias in the selection of reported results as there were no pre-published protocols for these studies.

#### Study characteristics and results

The study characteristics of the 16 observational studies included are shown in Table S2. These studies investigated a number of different populations including: paediatric critically ill patients,^39^ critically ill patients,^36,37,40–45^ critically ill patients with alternate renal functions such as receiving continuous RRT,^46^ or augmented renal clearance,^47^ patients treated for specific infections,^48,49^ and burns patients.^38,50^ The majority of studies (11/16, 69%) included non-penicillin antibiotics with analyses not separating outcomes for individual antibiotics.^36–39,41,42,44–46,49,50^ For instance, the study carried out by Machado *et al.*,^50^ which showed no difference in clinical outcomes between patients managed with TDM compared with patients who were not, looked at TDM of piperacillin, imipenem, meropenem, and vancomycin; piperacillin represented just 11.7% of the TDM measurements.

Another common feature (in 9/16 studies, 56%) was the inclusion of concomitant antibiotics in the regimen, with the majority having no analysis of the impact of this on clinical outcomes.^36,39,40,42,47–49,51^

A key component of TDM is adjustment of doses to improve target drug level attainment and this was shown to be successful in a number of studies.^38–40,43,46,49^ However, in others the effectiveness of dose adjustment was either not assessed^37,45,47,50^ or was shown to be ineffective.^36,41,42,44,48,51^

The study carried out by Richter *et al.*^40^ (*n *= 484) included data from the largest number of patients and looked at the effects of TDM-guided continuous infusions of piperacillin on PK/PD outcomes and analysed mortality data in relation to piperacillin concentration as a secondary outcome. By comparing target attainment before and after antibiotic measurement and dose adjustment, the study showed that their TDM protocol was effective at significantly improving target attainment. A U-shaped mortality rate over different concentrations of piperacillin was found with mortality rates increasing when patients had subtherapeutic concentrations (≤32 mg/L) and supratherapeutic concentrations (≥65 mg/L), with the highest mortality rates seen in patients with piperacillin concentrations >100 mg/L.^40^ This study concluded that TDM may improve clinical outcomes of patients undergoing continuously infused piperacillin treatment, and similar studies need to be carried out on other penicillins to identify whether or not this result applies across penicillins. Like the other studies there was a risk of bias associated with this study; the higher mortality seen in the groups with higher piperacillin concentrations may have been a product of the severity of infection. These groups (65–99 mg/L and >100 mg/L) had more patients experiencing septic shock, more patients needing RRT, with lower creatinine clearance. Additionally, the effect of the co-intervention (co-administered ciprofloxacin) was not assessed. A similar finding was seen in Scharf *et al.*,^45^ where mortality was significantly higher in patients with antibiotic concentrations above and below the target range (100% *fT *> 1–4* *×* *MIC). The study by Al-Shaer *et al.*,^44^ also included a large patient pool and showed significant associations between achieving target outcomes and clinical cure, microbial resolution, and suppression of resistance. Further, they showed that measuring concentrations early in the treatment was significantly associated with shorter ICU stay, clinical success, and lower mortality. SOFA score was also associated with clinical success, ICU length of stay, and mortality. The majority of the patients were treated with cephalosporins and only 24% were treated with penicillins.^44^

### Case series and case studies

#### Quality assessment

Due to the inherent high risk of bias in case studies, all of the case studies and case series scored ‘Definitely High’ using the OHAT risk of bias tool.^52^

#### Study characteristics and results

Table S3 shows the characteristics of the case series and case studies where TDM was used to influence penicillin administration for their treatment. Clinical success was seen in 9/12 patients where penicillin TDM was used. This relatively small number of case series and case studies cannot provide insight into whether TDM of penicillin antibiotics results in positive clinical outcomes. However, these studies show there is a large variation in how TDM is being carried out, on which patients, and that there is no standardized approach to TDM-guided treatment with penicillins.

### TDM implementation

The implementation of TDM was not consistent across the studies. Table 3 shows the different pharmacological targets used in the included studies; 16 different concentration targets were used across 28 studies. Further, factors that may directly affect the effectiveness of TDM such as: frequency of sampling, number of samples taken, dose adjustment protocols, were not standardized across studies.

**Table 3.** Pharmacological targets used for TDM of penicillins in patients

Pharmacological target	Reference
100% *fT* > MIC	^35–38,44,45,50,55–57^
50% *fT* > MIC	^35,36,58^
100% *fT* > 4–10 × MIC	^33,42^
40% *fT* > 4–6 × MIC	^39^
100% *fT* > 4–8 × MIC	^51^
100% *fT* > 1–10 × MIC	^46^
50% *fT* > 4 × MIC	^36^
100% *fT* > 4 × MIC	^36,38,41,44,45^
100% *fT* > 10 × MIC	^36^
100% *fT* > 4–5 × MIC	^42^
40% *fT* > MIC	^59,60^
100% *fT* > 4–5 × MIC	^61,62^
*T* > 2–4 × MIC	^43^
*T* > MIC	^49,63^
*fC*_ss_3–10 × MIC	^49^
Specific antibiotic concentration specified	^34,40,47,48^

## Discussion

There is little evidence to suggest that penicillin TDM is associated with improved health outcomes, but neither health outcomes nor reductions in emergence in AMR have been adequately addressed by currently published studies. There are few published RCTs that have evaluated the efficacy of TDM on health outcomes of patients treated with penicillins; the three RCTs identified here were designed to detect pharmacological outcomes and so were underpowered to detect an effect on clinical outcomes. Meta-analysis was considered but due to the high risk of bias, the degree of heterogeneity in TDM methodology, and variation in patient groups, this was not performed. The primary outcomes of the included observational studies were also pharmacologically based, but many of these studies could contribute little to the research question because of the lack of consideration of confounding factors in the analysis.^36–39,41,46,47,51^ Four papers did include an analysis of confounding factors, however, key factors were missed in three.^42,44,50^ Consequently, the results of these studies need to be interpreted with caution in terms of patient health outcomes.

Pharmacological targets were highly variable across the included studies (Table 3) highlighting the clinical uncertainty in this area. Studies similar to Richter *et al.*,^40^ who investigated clinical response to measured antibiotic concentration in humans, are needed to identify optimal therapeutic ranges. With results of such studies, standardized therapeutic targets based on clinical response in humans, can be followed. However, important confounding factors such as severity of infection need to be considered when interpreting results. If a patient has a severe infection they may be renally impaired, resulting in penicillin accumulation. As the condition of these patients is worse, they are more susceptible to clinical failure, this may result in penicillins appearing to have a toxic effect as high plasma concentrations will correlate to mortality. This may be the case in the study by Richter *et al*.,^40^ in which the groups with higher than target and extremely high piperacillin concentrations had lower rates of creatinine clearance, higher need for RRT, and more instances of septic shock compared with the groups that hit the target, or had lower than target levels.

The effectiveness of the dosing adjustment protocol to improve target attainment was variable across the studies. Target attainment was shown to be improved, subsequent to dosing adjustments, in some studies,^33,35,38–40,43,46,49^ but not in others,^34,36,41,42,44,48,51^ or the effectiveness of dose adjustment was not considered.^37,45,47,50^ To ensure the success of a TDM intervention, dose adjustment protocols need to alter the dosing regimen sufficiently to improve target attainment. If the effectiveness of dose adjustment protocols to improve target attainment is not assessed, or is ineffective, the resulting TDM practice will be sub-optimal. Since the PK/PD of penicillins is so variable, and subject to physiological changes such as renal function, TDM protocols are needed that are effective at changing penicillin concentrations in patients and that assess penicillin concentrations regularly enough to ensure optimal concentrations throughout treatment.

A number of health outcomes were inconclusively assessed including mortality,^33^ infection resolution,^33,34^ and fever duration;^35^ studies that included a comparator group have been presented in Table 4. Some studies used subjective measures of clinical outcomes which are unhelpful because of the high risk of bias, particularly in the context of retrospective, unblinded analyses. The majority of studies showed little clinical improvement upon implementation of TDM.^33–35^ However, the study that included the largest number of patients, as well as showing that the dose adjustment was successful at improving target attainment, found a significant association between piperacillin concentration and mortality rates, when using TDM to influence continuous infusion dosing.^40^ This suggests that TDM has the potential to optimize drug concentration and improve mortality, however, severity of illness needs to also be taken into account. Patients who had piperacillin concentrations above the target, in whom mortality was highest, also had higher levels of septic shock, more need of RRT, and lower creatinine clearance, which suggests these patients had more severe infections. A regression analysis between health outcomes and a severity of infection measure (APACHE-II score, SOFA score etc.) was not performed.^40^ The study by Al-Shaer *et al.*^44^ looked at clinical outcomes associated with measured β-lactam concentrations and showed that clinical outcomes were significantly associated with achieving target concentrations. Target attainment of *fT*_>MIC_ and *fT*_>4×MIC_ were associated with clinical cure, and microbial resolution. Additionally, the study saw significant associations between the time it takes to measure drug concentration and clinical failure, higher mortality, and a longer stay in ICU.^44^ This highlights the importance of taking TDM measurements as soon as possible. Despite this, confounding factors need consideration and SOFA score was also associated with clinical cure, ICU length of stay, and mortality. As well as associations with improved clinical outcomes, Al-Shaer *et al.*^44^ showed that target attainment was also associated with suppression of resistance, another driver of this systematic review. However, the impact of TDM on the emergence of AMR was not assessed by any of the RCTs and was only mentioned in one other observational study,^47^ where it was observed that the three clinical failures in the study were all a result of acquisition of secondary resistance to piperacillin/tazobactam.^47^ Theoretically, penicillin treatment guided by TDM has the potential to reduce AMR. A recent systematic review, investigating the antibiotic concentrations required to suppress AMR in Gram-negative bacteria, showed a β-lactam *C*_min_/MIC ratio of ≤4 can result in the emergence of AMR.^11^ Results from Al-Shaer *et al.*^44^ could be a promising sign that AMR emergence could be tackled through TDM. However more studies that assess the association of penicillin concentration and the emergence AMR in humans are needed as the majority of patients in that study received other β-lactams.

**Table 4.** Effect of TDM of penicillins on health outcomes

Reference (study location)	Population	Effective dose adjustment protocol?	Bacterial persistence	Mortality	In-hospital stay	Length of stay on ICU	Acute kidney injury	Toxicity or adverse effects	Treatment efficacy	Antimicrobial resistance
Randomized controlled trials										
Sime *et al.* 2015. (Adelaide, Australia)^35^	Febrile neutropenic patients with haematological malignancies, *n* = 32	Target attainment significantly improved with TDM compared to the standard-care control.	—	—	—	—	—	—	NSD	—
Fournier *et al.* 2018. (Lausanne, Switzerland)^34^	Burns patients, *n* = 30	Target attainment slightly improved with TDM compared to the standard-care control.	—	—	—	—	—	—	NSD	—
De Waele *et al.* 2013. (Ghent, Belgium)^33^	Non-renally impaired patients, *n* = 28	Target attainment significantly improved with TDM compared to the standard-care control.	NSD	NSD	—	—	—	—	NSD	—
Non-randomized observational studies with comparator group										
Machado *et al.* 2017. (São Paulo, Brazil)^50^	Burns patients, *n* = 16	Not specified	—	NSD	—	—	—	—	NSD	—

Abbreviations: –, not assessed; NSD, no significant difference (with TDM).

This systematic review has a number of limitations. Firstly, a number of studies reported outcomes that included results from non-penicillin antibiotics, outcomes were not reported for the individual antibiotics. Secondly, of the included studies all of the RCTs and the majority of the observational studies reported clinical outcomes as secondary aims. Particularly in the observational studies, this resulted in a bias for confounding factors. Thirdly, there was lots of variation in the way TDM of penicillins was implemented, the patient populations that it was carried out with and the outcomes that were reported. This makes it challenging to compare studies, and is why a meta-analysis was inappropriate.

This systematic review found no statistically significant evidence that penicillin TDM can improve health outcomes, when comparing patients treated with TDM and standard care, and no evidence that it was detrimental to health outcomes. The processes and practice of penicillin TDM were highly variable. The included studies were designed to assess pharmacological primary outcomes and patient health outcomes were secondary, hence studies were generally underpowered for patient health outcomes. Observational studies demonstrated that TDM can be effectively delivered and can be associated with good outcomes. Additional studies are needed to assess optimal TDM conditions for penicillin antibiotics to drive a standardized approach. These studies need to be powered to elucidate the impact of TDM of penicillins on health outcomes, and the emergence of AMR. Two protocols of RCTs investigating clinical outcomes in piperacillin/tazobactam^53^ and β-lactams^3^ have been published, hopefully these trials will help clarify the effect TDM of penicillins has on patient, and public, health.

## Supplementary Material

dkac101_Supplementary_Data

## Supplementary data

Search details and Tables S1 to S3 are available as Supplementary data at *JAC* Online.
