# Supplementary material for: A systematic review of the effect of therapeutic drug monitoring on patient health outcomes during treatment with penicillins
Source: J Antimicrob Chemother. 2022 Mar 31;77(6):1532–41. doi: 10.1093/jac/dkac101 (PMC9155611; doi:10.1093/jac/dkac101)
Supplement: dkac101_Supplementary_Data [file dkac101_supplementary_data.docx]

Supplementary data

Supplementary Material: Search details

Ovid MEDLINE(R) ALL 1946 to September 24, 2021

Searched: 24 Sep 2021

1 exp Penicillins/ (82322)

2 (phenethicillin* or phenoxymethylpenicillin* or phenoxymethyl-penicillin* or benzylpenicillin* or benzyl-penicillin* or cloxacillin* or flucloxacillin* or methicillin* or amoxicillin* or ampicillin* or ciclacillin* or mezlocillin* or pivampicillin* or carbenicillin* or bacampicillin* or talampicillin* or temocillin* or piperacillin* or azlocillin* or ticarcillin* or carfecillin* or Mecillinam* or pivmecillinam* or co-amoxiclav* or coamoxiclav* or co-fluampicil* or Cofluampicil*).tw. (87706)

3 1 or 2 (143067)

4 exp Drug Monitoring/ (22362)

5 (TDM or drug monitoring).tw. (11938)

6 (dos* adjustment* or dos* alteration* or dos* escalation*).tw. (23235)

7 (personal* adj2 (medicine or therap* or treatment* or dos*)).tw. (30894)

8 (custom* adj2 (medicine or therap* or treatment* or dos*)).tw. (1817)

9 (individual* adj2 (medicine or therap* or treatment* or dos*)).tw. (43602)

10 4 or 5 or 6 or 7 or 8 or 9 (124249)

11 3 and 10 (791)

12 limit 11 to yr=”2020 -Current” (130)

Embase Classic+Embase 1947 to 2021 September 24

Searched: 24 Sep 2021

1 exp penicillins/ or exp penicillin derivative/ (356617)

2 (phenethicillin* or phenoxymethylpenicillin* or phenoxymethyl-penicillin* or benzylpenicillin* or benzyl-penicillin* or cloxacillin* or flucloxacillin* or methicillin* or amoxicillin* or ampicillin* or ciclacillin* or mezlocillin* or pivampicillin* or carbenicillin* or bacampicillin* or talampicillin* or temocillin* or piperacillin* or azlocillin* or ticarcillin* or carfecillin* or Mecillinam* or pivmecillinam* or co-amoxiclav* or coamoxiclav* or co-fluampicil* or Cofluampicil*).tw. (120598)

3 1 or 2 (394368)

4 exp drug monitoring/ (56297)

5 (TDM or drug monitoring).tw. (18475)

6 (dos* adjustment* or dos* alteration* or dos* escalation*).tw. (47935)

7 (personal* adj2 (medicine or therap* or treatment* or dos*)).tw. (47341)

8 (custom* adj2 (medicine or therap* or treatment* or dos*)).tw. (2896)

9 (individual* adj2 (medicine or therap* or treatment* or dos*)).tw. (67109)

10 4 or 5 or 6 or 7 or 8 or 9 (219220)

11 3 and 10 (3374)

12 exp animals/ not exp human/ (5631269)

13 exp nonhuman/ not exp human/ (4860096)

14 exp experimental animal/ (764465)

15 exp veterinary medicine/ (58795)

16 animal experiment/ (2728771)

17 or/12-16 (8079089)

18 11 not 17 (3125)

19 limit 18 to yr=”2020 -Current” (366)

Search Name:

Date Run: 26/10/2020 11:38:26

Comment:

ID Search Hits

#1 MeSH descriptor: [Penicillins] explode all trees (5771)

#2 (phenethicillin* or phenoxymethylpenicillin* or phenoxymethyl-penicillin* or benzylpenicillin* or benzylpenicillin* or cloxacillin* or flucloxacillin* or methicillin* or amoxicillin* or ampicillin* or ciclacillin* or mezlocillin* or pivampicillin* or carbenicillin* or bacampicillin* or talampicillin* or temocillin* or piperacillin* or azlocillin* or

ticarcillin* or carfecillin* or Mecillinam* or pivmecillinam* or co-amoxiclav* or coamoxiclav* or co-fluampicil* or Cofluampicil*):ti,ab,kw (10982)

#3 #1 or #2 (11809)

#4 MeSH descriptor: [Drug Monitoring] explode all trees (1845)

#5 ("drug monitoring" or TDM):kw,ti,ab (3399)

#6 ("dose adjustment*" or "dose escalation*" or "dose alteration*"):kw,ti,ab (10027)

#7 ((personal* or custom* or individuali?ed*) NEAR/2 (medicine or therap* or treatment* or dos*)):ti,ab,kw (5346)

#8 #4 or #5 or #6 or #7 (18488)

#9 #3 and #8 (109)

Published in 2020-2021 (10)

Web of Science 24Sep21

CORE collection

# 1 TS=(penicillin?) (7622)

# 2 TS=(phenethicillin*) or TS=(phenoxymethylpenicillin*) or TS=(phenoxymethyl-penicillin*) or TS=(benzylpenicillin*) or TS=(benzyl-penicillin*) or TS=(cloxacillin*) or TS=(flucloxacillin*) or TS=(methicillin*) or TS=(amoxicillin*) or TS=(ampicillin*) or TS=(ciclacillin*) or TS=(mezlocillin*) or TS=(pivampicillin*) or TS=(carbenicillin*) or TS=(bacampicillin*) or TS=(talampicillin*) or TS=(temocillin*) or TS=(piperacillin*) or TS=(azlocillin*) or TS=(ticarcillin*) or TS=(carfecillin*) or TS=(Mecillinam*) or TS=(pivmecillinam*) or TS=(coamoxiclav*) or TS=(coamoxiclav*) or TS=(co-fluampicil*) or TS=(Cofluampicil*) (89886)

# 3 #1 or #2 (95503)

# 4 TS=("drug monitoring") or TS=(TDM) (19619)

# 5 TS=("dos* adjustment*") or TS=("dos* escalation*") or TS=("dos* alteration") (28481)

# 6 TS=(personal* NEAR/2 (medicine or Therap* or treatment* or dos*)) (43755)

# 7 TS=(custom* NEAR/2 (medicine or Therap* or treatment* or dos*)) (2672)

# 8 TS=(individual* NEAR/2 (medicine or Therap* or treatment* or dos*)) (63086)

# 9 #4 or #5 or #6 or #7 or #8 (151165)

# 10 #3 and #10 (803)

Following the database search a hand search was carried out. Studies were identified through searching the reference lists of relevant reviews of the topic.

Table S1 – Study characteristics of RCTs assessing the role of TDM during therapy with penicillin class antibiotics.

| **Reference** | **Population** | **Penicillin** | **TDM measurement** | **Concomitant Antibiotics** | **Renal Function** | **Pathogen and MIC** | **Pharmacological Target** | **Pharmacological Target Attainment** | **Microbiological Resolution** | **Clinical Resolution** | **Emergence of Antimicrobial Resistance** |
| --- | --- | --- | --- | --- | --- | --- | --- | --- | --- | --- | --- |
| Sime et al. 2015. Adelaide, Australia ^1^.  RCT measuring PK/PD and clinical differences between patients where dosing regimens were adjusted according to TDM over 3 days, and patients with empiric therapy | Febrile neutropenic patients with haematological malignancies (n=32)  Condition – febrile neutropenia – defined according to published definitions. | Piperacillin-Tazobactam, n=32  4.5g every 6 or 8 hours | Total plasma antibiotic concentrations were determined though LC-MS/MS. Unbound concentrations were calculated assuming 30% protein binding.  Sampling for TDM measurement was done over three days. Two samples were taken on days 1 and 2, one at 50% of the dosing interval and one at trough level, 15 mins before the next dose. The first sample was taken during the third or fourth dosing interval during steady state. One trough sample was taken for day 3. | Gentamicin was co-administered in 30/32 (94%) patients, 15/16 patients in both the intervention and control group. In the intervention group vancomycin (n=3), trimethoprim/sulphamethoxazole (n=1) and metronidazole (n=1) was also co-administered. In the control group, vancomycin (n=2), ciprofloxacin (n=2), and trimethoprim/sulphamethoxazole (n=1) was also co-administered.  Concomitantly administered antibiotics were balanced across both groups. | On days 1, 2, and 3 of TDM the intervention group had a CrCl (ml/min/1.73m^2^, median (IQR)) of 80 (70-121), 86 (68-131), and 86 (66-123), respectively. Comparatively, the control group, on days 1, 2, and 3, had a CrCl of 92 (74-133), 98 (70-129), and 90 (70-120).  ARC, defined as CrCl >130 ml/min/1.73m­^2^, was present in 5 patients in both groups. | Infecting pathogens were isolated in 13/32 patients. Intervention group: *Bacillus cereus* (n=1), *Escherichia coli* (n=2), *Enterobacter cloacae* (n=1), *Staphylococcus* spp. (n=1), viridans streptococci (n=1). Control group: *Bacillus cereus* (n=1), *Escherichia coli* (n=1), *Klebsiella pneumoniae* (n=1), *Staphylococcus* spp. (n=2), viridans streptococci (n=2).  Local susceptibility data, measured MIC or EUCAST data were used when culture results were available. Where specified organisms had not been identified, the highest EUCASET clinical breakpoint for likely pathogens were selected. | The primary pharmacological target was 100% ƒT>MIC, a secondary target of 50% ƒT>MIC was also used.  Dose adjustments were made when concentrations were below the MIC value. If TDM results were within 20% of the MIC dosing frequency was increased by 25%-50%, or by extending the infusion time. If TDM result was >20% below the MIC, frequency of dosing was increased and infusion time extended.  If TDM result was >10x MIC then the dosing frequency was decreased by 25%-50% or the dose was decreased by 50%. | Target attainment significantly improved with TDM compared to controls.  Three TDM measurements took place over three days. In the first TDM, 31% and 25% of the control group met the 50% and 100% ƒT>MIC target, compared to 44% and 19% of the intervention group, [P=0.716] and [P=1.00], respectively.  On the second TDM, 31% and 19% of the control group met the 50% and 100% target, compared to 94% and 69% of the intervention group, [P=0.001], and [P=0.012], respectively.  On the third TDM 7% of the control group met the 100% target, compared to 73% of the intervention group, [P=0.004].  The presence of ARC resulted in significantly lower antibiotic concentrations [P<0.01]. | Not specified | There were no differences in clinical outcomes between the two groups. In both groups, the median duration of fever was the same, 2 days (intervention IQR: 1-3 days, control IQR: 1-4 days).  There was also no statistical differences in duration of neutropenia: intervention group: 6 days (3-8 days); control group: 6 days (4-13 days). | Not specified |
| Fournier et al. 2018. Lausanne, Switzerland ^2^.  RCT evaluating the impact of therapy guided by TDM has on target attainment in burns patients, clinical outcomes were also reported. | Burns patients receiving intravenous antibiotics for a range of infections, mainly pneumonia (24) and skin/soft tissue infection (7) (n=38)  Patients receiving penicillins n=30  (N.B. Study also included non-penicillin antibiotics)  Intervention group median SAPS II score: 31.0 (IQR: 22.0-42.0).  Control group median SAPS II score: 28.0 (IQR: 20.0-45.0) | Amoxicillin, n=4,  empiric dose: 1g q6h/2g 4h  Amoxicillin-clavulanic acid, n=19,  empiric dose:  1.2g-2.2g q4-6h  Flucloxacillin, n=2,  empiric dose:  2g q4-6h  Piperacillin-tazobactam, n=11,  empiric dose: 4.5g q6-8h | Total serum antibiotic concentrations were measured every other day using HPLC-MS/MS. Unbound concentrations were calculated using published protein binding data.  Samples were taken every two days for TDM measurement. | Not specified | Not specified | Most commonly isolated pathogens were *S. pneumoniae*, *S. aureus, Pseudomonas aeruginosa* and *Enterobacteriales.* | The predefined pharmacological trough target was set by antibiotic:  - Amoxicillin:  Min level: 8 mg/L  Max level: 40 mg/L  - Flucloxacillin:  Min level: 40 mg/L  Max level: 200 mg/L  - Piperacillin:  Min level: 8 mg/L  Max level: Unknown (30 mg/L)  In the intervention group dose was adjusted by increasing or decreasing dose amount or frequency of dose, or by adjusting infusion duration. In the standard-care group clinicians could request a rescue TDM result in special cases. | Of initial TDM results in the intervention group, of penicillins used to treat infection episodes: 13 (68.4%) were outside the target range, 10 (52.6%) were too low and 3 (15.8%) were too high. After intervention, subsequent TDM results, 11 were outside the target range (44%), with 9 (36%), and 2 (8%) being too high.  Of initial TDM results, of penicillins used to treat infection episodes, in the standard care group: 9 (45%) were outside the target range, 7 (35%) were too low, and 2 (10%) were too high. Of subsequent TDM results, where the clinicians were blinded to results, 19 (45%) were outside the target range, with 15 (35.7%) being too low and 4 (9.5%) being too high. | Not specified | No difference was seen in clinical outcomes between the two groups. There was 33/36 (91.7%) successfully treated episodes of infection in the intervention group, compared to 30/31 (96.8%) of infection in the standard-of-care group.** | Not specified |
| De Waele et al. 2013. Ghent, Belgium. ^3^  RCT evaluating the impact of altering dosing regimens according to TDM in non-renally impaired patients receiving piperacillin and meropenem. | Non-renally impaired patients receiving piperacillin (and meropenem) n=41 **  Intervention group median APACHE II score: 19 (IQR:12-24)  Control group median APACHE II score: 17 (IQR: 13-23) [P=0.557] | Piperacillin-Tazobactam, n=28  Extended infusion 4g infused over 3 hours every 6 hours  (Meropenem, n=13  Extended infusion 1g infused over 3 hours every 8 hours)** | Daily total antibiotic concentrations were determined through HPLC-MS/MS.  Unbound antibiotic concentrations were not determined, total amounts used.  Samples were taken daily. The first sample was taken after a minimum of 3 infusions were completed to reach steady-state. Samples were taken mid-dosing interval and prior to the next dose during the first 3 days of dosing. During the final 4 days of dosing only trough samples were taken. | Not specified | Renally impaired patients (eGFR <80mL/min) were excluded from the study.  Intervention group day 1 median creatinine clearance was 130 mL/min (IQR: 92-177 mL/min), day 3 median creatinine clearance was 155 mL/min (IQR: 83-182 mL/min).  Standard care group day 1 median creatinine clearance was 108 mL/min (IQR: 88-145 mL/min) [P=0.291], day 3 median creatinine clearance was 110 mL/min (IQR: 90-165 mL/min) [P=0.697]. | The most commonly isolated bacterial pathogens were *E. coli* (n=7), *Klebsiella pneumoniae* (n=7), and *Pseudomonas aeruginosa* (n=6). There was a total of 43 causative pathogens isolated from 27 patients. The median MIC for piperacillin was 2mg/L (IQR: 1.5-8mg/L). | The pharmacokinetic targets were 100% 𝑓T>4-10xMIC and 100% 𝑓T>MIC was used in the analysis.  If TDM result was <4xMIC then piperacillin dosing frequency was increase.  If TDM result was >10xMIC dosing frequency was decreased, if subsequent TDM result was still above 10xMIC then the dose was reduced. | Initially in the intervention group there was a PK target attainment of 9% and 68% for 100% 𝑓T>4xMIC and 100% 𝑓T>MIC targets, respectively. Compared to the control group which had a target attainment of 20% and 70% for the targets 100% 𝑓T>4xMIC and 100% 𝑓T>MIC, respectively.  After 72 hours, in the intervention group there was 100% 𝑓T>4xMIC target attainment of 58% versus 16% in the standard care group (P=0.007). For the 100% 𝑓T>MIC target, there was a 95% target attainment in the intervention group compared to a 68% target attainment in the control group (P=0.045).  TDM significantly improved PK target attainment. ** | In the intervention group there was bacterial persistence at day 7 in 1 patient. In the control group there was bacterial persistence in 5 patients (P=0.09). | In the intervention group clinical failure was seen in 2 patients, compared to 4 in the control group (P=0.41).** Median SOFA scores in the intervention group changed from 5.5 at the baseline to 3 at day 7 (P=0.093). In the control group the median SOFA score change was from 5 to 4. from baseline to day 7 (P=0.575).**  In the intervention group 6.7% (n=1) of patients died in the ICU compared to 30.8% (n=4) in the control group. In the intervention group 13.3% (n=2) of patients died within 28-days compared to 30.8% (n=4) in the control group. | Not specified |

Abbreviations: APACHE II, Acute Physiology and Chronic Health Evaluation; ARC, Augmented Renal Clearance; CrCl, Creatinine clearance; eGFR, estimated Glomerular Filtration Rate; EUCAST, European Committee on Antimicrobial Susceptibility Testing; 𝑓T>MIC, time unbound antibiotic concentration was greater than the MIC; IQR, Interquartile Range; (HP)LC-MS/MS, (High-Performance) Liquid Chromatography coupled tandem Mass Spectrometry; MIC, Minimum Inhibitory Concentration; PK/PD, Pharmacokinetic/Pharmacodynamic; RCT, Randomised Controlled Trial; SAPS II, Simplified Acute Physiology Score; TDM, Therapeutic Drug Monitoring; **, Includes data from non-penicillin antibiotics.

Table S2 – Study characteristics of observational studies

| **Reference** | **Population** | **Penicillin** | **TDM measurement** | **Concomitant Antibiotics** | **Renal Function** | **Pathogen and MIC** | **Pharmacological Target** | **Pharmacological Target Attainment** | **Microbiological Resolution** | **Clinical Resolution** | **Emergence of Antimicrobial Resistance** |
| --- | --- | --- | --- | --- | --- | --- | --- | --- | --- | --- | --- |
| Cies et al. 2018. Philadelphia, USA.  ^4^  Observational study investigating dosing regimen effectiveness and the effect of TDM on clinical outcomes.  Study divided patients into two cohorts, the total cohort, comprising all included patients, and the infected cohort, those where a pathogen was microbiologically isolated. | Paediatric critically ill patients and PICU patients receiving extracorporeal membrane oxygenation or CRRT (n=18)  Total cohort:  Median Pediatric RISk of Mortality score: 17 (range: 2-39)  Confirmed infection cohort:  Median Pediatric RISk of Mortality score: 21 (range: 5-32) | Ampicillin  Piperacillin-Tazobactam  ** n=64 non-penicillin antibiotics | Total plasma antibiotic concentrations were determined by LC-MS/MS, the free fraction was estimated using published protein binding data.  At least two samples were obtained per patient.  Intermittent dosing (30-60 min infusion):  Samples were taken within 20 minutes of end of infusion to up to 2 hours after end of infusion. Second samples were taken after at least 1 hour.  Extended infusion (3-4hr infusion): Samples were taken at the end of infusion, and 1hr after first sample.  Continuous infusion: Samples taken after at least 8 hrs. | Concomitant antibiotics, such as vancomycin, gentamicin, and tobramycin , were administered.  There was no analysis of the effect of concomitant antibiotics on outcome, nor any reporting of concomitant antibiotic administration between cohorts. | Not specified. | Microbiological infection was confirmed, through isolation of the infecting pathogen in 23/82 patients.  Isolated pathogens treated with piperacillin-tazobactam:  *Pseudomonas aeruginosa* (n=4, MIC: 16mg/L); *Escherichia coli* (n=3, MIC: 4mg/L); *Acinetobacter* spp. (n=2, MIC: 8, 16 mg/L); *Klebsiella pneumoniae* (n=1, MIC: 4 mg/L).  In patients where an organism was not isolated then the highest MIC breakpoint value from the Clinical and Laboratory Standards Institute (CLSI) was used, Piperacillin: 16 mg/L, Ampicillin: 8 mg/L | 40% ƒT>4-6xMIC  Dosing frequency was prolonged if TDM result was too high and shortened if it was too low, or infusion times lengthened. | Across the total cohort 95% (78/82) of treatments in patients failed to reach the 40% fT>4-6xMIC target. **  Of the 78 patients with treatments outside the target range 5 (6.4%) had a concentration >6xMIC, and 73 (93.6%) had a concentration <4xMIC. **  In patients with an isolated pathogen there was no patients with a dosing regimen that met the pharmacological target. **  In patients where the target was not met dosing regimens were altered to meet the pharmacological target. Improvement in target attainment not specified ** | In patients where a pathogen was isolated, there was a positive microbiological response in 100% of cases. ** | In patients where a pathogen was isolated, there was a positive clinical response of 95.7% (22/23), resulting in a mortality rate of 4.3%. The mortality rate of the total cohort was 12.2%.  No statistically significant association between target attainment and clinical response or mortality was reported. | Not specified |
| Duszynska et al. 2012. Wroclaw, Poland. ^5^  Study assessing using TDM to adjust continuous infusions to meet target serum concentrations in VAP patients. | VAP patients (n=16)  Median SOFA score: 8 (range: 2-16).  Median APACHE II score: 26.5 (9-35). | Piperacillin-Tazobactam  Initially a continuous infusion of 10g daily. | Total serum piperacillin concentrations were measured by HPLC.  Samples for analysis were taken before piperacillin administration and at 1, 6, 12, 24, 48, 72, and 96 hours. Samples from 48, 72, and 96 hours were used for dose adjustment, for antibiotic to reach steady state. | Concomitant antibiotics were included in some patients.  Netilmicin n=3, vancomycin m=1, trimethoprim/sulphamethoxazole n=1, piperacillin monotherapy n=11.  Effects of concomitant antibiotics on outcomes not reported. | Serum creatinine 1.6 +/- 1.2 mg/dL  CrCl: 69 +/- 48 mL/min  Effects of renal function on target attainment not reported. | Pathogens from patients with adequate antibiotic concentrations at all times: MSSA n=4, *Enterococcus faecalis* n=2, others n=8. Median MIC: 1mg/L, IQR: 0.425 - 1mg/L  Pathogens from patients with inadequate concentrations at times: *Pseudomonas aeruginosa* n=4, others n=3. Median MIC: 24 mg/L, IQR: 12-28 mg/L.  MIC values determined by Etest. | 100% T>4-8xMIC.  If TDM result showed concentration outside of target range doses were increased or decreased by 2g. | 10/16 patients had adequate piperacillin concentrations at all times. 6/10 had inadequate concentrations at some point.  Dosing regimens were reduced in 7 patients, and increased in 6. Of the 6 where dosing regimens were increased, serum concentrations reached target levels for one additional pathogen. | Microbiological eradication was seen in 13 strains (62%), and presumed in 2 other strains. Persistent colonisation was present in only one patient, this patient experienced inadequate piperacillin concentrations at times. | Across all included patients 5/16 patients died, 2/10 (20%) died of those where piperacillin concentrations were always adequate, 3/6 (50%) died  of patients which experienced inadequate concentrations.  Of patients which had an adequate piperacillin concentration at all times, clinical success was seen in 9/10. Of patients that had an inadequate measured concentration at some time, there was clinical success in 3/6.  No statistical associations were made between achieved adequate concentrations and clinical outcomes. | Not specified |
| Economou et al. 2017. Brisbane, Australia. ^6^  Study describing how dosing regimens guided by TDM effects patients receiving CRRT. | Patients undergoing continuous renal replacement therapy (CRRT) n=76  Patient condition not specified. | Piperacillin-Tazobactam  4.5g q6-12h  Ampicillin  1g q6h  Penicillin G  1.2-1.8g q4-6h  Flucloxacillin  1-2g q4-8h  **Study also included non-penicillin antibiotics ** | Total and unbound serum concentrations of antibiotic were measured by HPLC. Samples were taken after 4 doses and prior to the next dose to determine trough concentrations at steady-state. Frequency of TDM measurement was not specified. | Concomitant antibiotics not specified. | Mean serum creatinine: 205.2 µmol/L (range: 36-711 µmol/L).  CVVHDF settings:  Mean +/- SD  *Q_b_* (mL/min): 192 +/- 22 (range: 150-250)  Mean +/- SD *Q_d_* (mL/min): 346 +/- 524 (range: 1000-3000)  Mean +/-SD *Q_f_* (mL/min): 144 +/- 110 (range: 0-1000)  Mean +/- SD Post-dilution rate (mL/h): 1532 +/- 739 (range: 50-3375)  Mean +/- SD Pre-dilution rate (mL/h): 1222 +/- 441 (range: 1000-2000).  No analysis of effects of CVVHDF on target attainment was carried out. | Most commonly identified pathogens: MSSA (n=11), *Klebsiella pneumoniae* (n=6), *Escherichia coli* (n=14), *Pseudomonas aeruginosa* (n=3), *Enterococcus faecalis* (n=4).  73% (n=81) of microorganisms were identified.  Where possible MIC values determined through VITEK2, otherwise EUCAST susceptibility breakpoints were used. The highest susceptible MIC was used from potential pathogens was used when no microorganism was isolated. | 100% fT>MIC – 100% fT≤10xMIC  When a TDM result showed a concentration outside of this range then the doses were accordingly increased or decreased. Specific dose adjustment protocol not specified. | Dose adjustment in 35% of patients, 24% of adjustments needing a decrease **  In piperacillin patients TDM results, 35 dosing regimens (66%) were maintained, 3 (6%) were increased and 15 (28%) were decreased.  In ampicillin patients TDM results, 1 dosing regimen (100%) was decreased.  In penicillin G patients TDM result, 1 (20%) dosing regimens was maintained, 1 (20%) was increased and 3 (60%) was decreased.  In flucloxacillin patients TDM results, 7 (100%) dosing regimens were maintained. | Not specified | 10 patients receiving penicillin antibiotics died.  9 patients were receiving piperacillin, with a median identified MIC of 8mg/L (IQR: 6.5-8 mg/L). Median unbound trough concentrations for patients that died (fC_min_): >10x MIC (IQR: 5x MIC - >10xMIC).  1 patient received flucloxacillin, with a identified MIC of 1 mg/L. Unbound trough concentration of flucloxacillin was >10x MIC.  No statistical associations were made between achieved adequate concentrations and clinical outcomes. | Not specified |
| Besnard et al. 2019. France. ^7^  Retrospective analysis of increased piperacillin-tazobactam concentrations in ARC patients. | Critically ill patients with ARC (n=35)  Median SAPS II: 42 (IQR:34-51)  Median modified SOFA score: 3 (IQR:1-6). | Piperacillin-Tazobactam  20g daily dose of piperacillin, after 4g 60 min infusion loading dose. | Method of antibiotic quantification not specified. Unbound concentrations were estimated by protein binding data. | Concomitant antibiotics administered across group, n=8 (23%) received either aminoglycosides or quinolone.  Analysis on the effects of concomitant antibiotics on outcome not reported. | CrCl on day of TDM measurement: 166 mL/min (159-191 mL/min).  Analysis of effects of renal function on target attainment not performed. | Enterobacteriaceae (n=33), *Staphylococcus* spp. (n=18), *Haemophilus influenzae* (n=8), Non-fermenting GNB (n=3), other (n=3), polymicrobial infection (n=20)  MIC values for individual infections not determined. MIC value used was the EUCAST clinical breakpoint for *Pseudomonas aeruginosa* (16 mg/L). | 100% ƒT>MIC – 100% ƒT>9.4xMIC  Underdosing was considered any unbound concentration below 16 mg/L, excessive dosing was considered any unbound concentration above 150 mg/L.  Dosing regimens were guided by TDM between 24hrs and 72hrs of antibiotic treatment. | 100% of patients met target piperacillin concentrations (>16 mg/L)  Median unbound piperacillin concentrations: 36.4mg/L, (27.7mg/L - 44.3mg/L).  No excessive concentrations were observed. | Not specified | Zero patients died during the study.  Clinical failure was observed in three patients.  No statistical associations were made between achieving adequate concentrations and clinical outcomes. | All three clinical failures were related to acquisition of secondary resistance to piperacillin-tazobactam.  No statistical associations were made between acquisition of secondary resistance and target concentration attainment. |
| McDonald et al. 2016. Australia. ^8^  Retrospective study looking at TDM guided beta-lactam dosing in critically ill patients and whether high-dose therapy results in toxicity. | Critically ill patients (n= 46)  Licensed-dose group:  Mean SOFA score: 3.5 (SD: +/- 2.1)  High-dose group:  Mean SOFA score: 3.7 (SD: +/- 2.7)  [P=0.81] | Piperacillin-Tazobactam  Patients categorised into licensed dose (≤16g/24h) and high dose groups (>16g/24h)  **Study also included non-penicillin antibiotics** | Unbound plasma concentrations were determined by HPLC.  Sampling was initiated 4 half-lives after start of therapy. Frequency of TDM was not reported. | Concomitant antibiotics not specified. | Piperacillin patients that received a licensed-dose CrCl: 95.6 mL/min +/-31.7 mL/min  Piperacillin patients receiving a high dose: CrCl: 108.4 mL/min +/-31.6 mL/min  [P=0.17].  Analysis of effects of renal function on target attainment not performed. | Most commonly identified pathogens in piperacillin patients: *Escherichia coli* (n=9), *Klebsiella pneumoniae* (n=6), *Staphylococcus aureus* (n=6)  Susceptibility data not specified.  Where pathogens were not determined MIC values were based on clinical breakpoint data of likely pathogens. | 100% ƒT>MIC  If TDM results showed low concentrations dosing frequency was increased by 25% - 50%. If the TDM result was within 20% of the target the method of infusion was extended, intermittent dosing to extended or continuous. If TDM result showed concentrations too high dosing frequency was reduced by 25% - 50%. | Piperacillin patients that received a high dose were prescribed significantly more antibiotic than the licensed dose group [P<0.001].  There was no difference in the number of treatment courses that achieved 100% fT>MIC. Licensed dose: n=10 (40%), high dose: n=12 (52.2%) [P=0.40]. | Microbiological control was seen in 39/46 patients, 18/23 in the licensed dose group and 21/23 in the high dose group [P=0.22].  No statistical associations were made between microbiological resolution and achieving adequate antibiotic concentrations. | In piperacillin patients there was no difference in the duration of therapy between the groups, licensed dose group: 5.9+/-3.7 days, high dose group: 6.7+/-3.6 days [P=0.45].  In piperacillin patients there was no difference in treatment failure [P=0.29], length of stay in ICU [P=0.52], length of hospital stay [P=0.93]. There were 2 deaths in the licensed dose group and 1 in the high dose group.  No excessive toxicity was seen in either group.  No statistical associations were made between achieving adequate concentrations and clinical outcomes | Not specified |
| Wong et al. 2018. Brisbane, Australia. ^9^  TDM guided dose adjustment in critically ill patients | Critically ill patients (n=237 infection episodes treated with penicillins)  Median APACHE II score: 22 (IQR: 16-27) ** | ‘n’ refers to number of infection episodes treated by penicillin  Ampicillin n=15 2g q6h  Penicillin G n=15 2.4g q4h  Flucloxacillin n=25 2g 4h  Piperacillin n=182 4.5g q8h  **Study also included non-penicillin antibiotics, n=132 infection episodes treated with non-penicillin antibiotics** | Unbound plasma concentrations were determined by HPLC-UV.  Sampling was undertaken after 4 prior doses, for PK to reach steady-state.  For intermittent dosing, a mid-point and trough sample was taken.  For continuous infusions, samples were not taken until four half-lives of the antibiotic had passed.  103 patients (31.2%) had multiple TDM measurements ** | Patients with concomitantly administered antibiotics were included, there was no analysis into the effect of concomitantly administered antibiotics on outcomes. | Serum creatinine concentration: 76 µmol/L (IQR: 53-129 µmol/L) **  Calculated CrCl: 101.5 mL/min (IQR: 9.1-163.0 mL/min) **  On day of sampling 68 patients (13.8%) were undergoing CRRT. **  ARC (CrCl >130 mL/min) was present in 192 patients (39.1%). **  The presence of ARC (CrCl >130 mL/min) was significantly associated with failure to achieve PK target (OR 2.47-3.33, P<0.05)**  Excessive antibiotic exposure was associated with decreased renal function (CrCl<50 mL/min, OR 9.12 (95% CI 3.05-27.25); P<0.01. CrCl 51-90 mL/min, OR 3.21 (95% CI 1.10-9.41); P=0.03).** | Pathogens responsible for infections not specified.  Of the culture-positive samples, MIC data was available for 12 samples. **  Where the MIC was not determined EUCAST clinical breakpoint values, for determined or suspected pathogens, were set as MIC values. Where no species was suspected or determined the highest MIC of a susceptible pathogen was selected. | Pharmacological targets of:  50% and 100% ƒT>MIC  50% and 100% ƒT>4xMIC  100% ƒT>10xMIC were set.  Where TDM results showed concentrations below 100% ƒT>MIC the frequency of dosing was increased by 25% - 50%. If TDM result was within 20% of target infusions were extended. Or continuous infusions were administered when antibiotic was at maximum dose according to products information.  When TDM result was >100% ƒT>10xMIC either the dose concentration was reduced by 50% or the dosing frequency was decreased by 25% - 50%. | Of patients where more than one TDM result was taken, there was no significant difference between initial TDM result and subsequent TDM result.  Of ampicillin administered cases, 53.3% achieved the 100% ƒT>MIC target, 33.3% achieved 100% ƒT>4xMIC, and 13.3% met the 100% ƒT>10xMIC limit.  Of penicillin G administered cases, 93.3% achieved the 100% ƒT>MIC target, 80.0% achieved 100% ƒT>4xMIC, and 80.0% met the 100% ƒT>10xMIC limit.  Of flucloxacillin administered cases, 52.0% achieved the 100% ƒT>MIC target, 32.0% achieved 100% ƒT>4xMIC, and 16.0% met the 100% ƒT>10xMIC limit.  Of piperacillin administered cases, 61.0% achieved the 100%ƒT>MIC target, 33.5% achieved 100% ƒT>4xMIC, and 13.2% met the 100% ƒT>10xMIC limit.  Extended infusions were associated with a decreasing probability of achieving 100% fT>MIC (OR 0.28 (95% CI 0.09-0.86); P=0.026) **  Excluding the 50% ƒT>MIC target, the type of antibiotic was significantly associated with target attainment, [P<0.01] ** | Not specified | There was a positive clinical solution in 71.2% of cases. **  There were 41 deaths across the study. **  There was a significant association between negative clinical outcome and an abdominal source of infection (OR 7.60, (95%CI 2.39-24.17; P=0.001). **  There was no association observed between patients that failed to achieve pharmacological targets and negative clinical outcomes in patients with a positive microbiological culture (100% fT>MIC: OR 0.88 (95% CI 0.40-1.94; P=0.74. 100% fT>4xMIC: OR 0.67(95% CI 0.29-1.55; P=0.35). ** | Not specified |
| Patel et al. 2012. Brisbane, Australia. ^10^  Study observing the effects of TDM guided dosing regimens in burns patients | Burns patients (n=48)  % Total body surface area (TBSA): 17 +/- 13 ** | Ampicillin n=2  2g q6h  Penicillin G n=8  2g q6h    Flucloxacillin n=24  2g q6h  Dicloxacillin n=8  2g q6h  Piperacillin-Tazobactam n=6  4.5g q6h  **The study also included patients receiving non-penicillin antibiotics n=2** | Plasma concentrations were determined by HPLC-UV. Unbound concentrations were estimated using published protein binding data.  Samples for TDM were taken after a minimum of prior doses, to ensure PK was at steady-state. Samples were taken within 15 minutes of next dose. | Concomitant antibiotics not specified. | Serum creatinine concentration over the whole study group: 86 µmol/L +/-30 µmol/L**  Analysis of effects of renal function on target attainment not performed. | Pathogens responsible for infections not specified.  Pathogen or suspected pathogen clinical breakpoint values from local antibiogram data were used for MIC values where possible, otherwise clinical breakpoint values from EUCAST data were used. | 100% ƒT>MIC and 100% ƒT>4xMIC were used as pharmacological targets.  If TDM results showed a concentration below the 100 ƒT>MIC target then dosing frequency was increased. If dosing frequency had already been increased then the infusion was extended. There was no protocol for antibiotic concentrations that were too high. | 30 patients (60%) received a dose adjustment after initial TDM result. Following dose adjustments all patients exceeded the minimum target**  In ampicillin patients 50% (n=1) reached the ƒT>MIC target, and 0 reached the ƒT>4xMIC target.  In penicillin G patients 25% (n=2), and 12% (n=1) of patients reached the ƒT>MIC, and ƒT>4xMIC targets, respectively.  In flucloxacillin patients 34% (n=8), and 16% (n=4) of patients reached the ƒT>MIC, and ƒT>4xMIC targets, respectively.  In dicloxacillin patients 0, and 25% (n=2) of patients reached the ƒT>MIC, and ƒT>4xMIC targets, respectively.  In piperacillin-tazobactam patients 0, and 34% (n=2) of patients reached the ƒT>MIC, and ƒT>4xMIC targets, respectively. | Not specified | A positive clinical outcome was seen in all patients included in the study.  In patients where the ƒT>MIC target was achieved there was a significantly shorter length of antibiotic treatment, 4.2 +/- 1.1 days, versus 5.3 +/-2.3 days [P=0.03], than in patients where the target was not met. **  When using the ƒT>4xMIC target there was not a statistically significant association, 4.1 +/- 1.1 days where the 4xMIC target was met versus 5.1 +/- 2.1 days; [P=0.17].** | Not specified |
| Roberts et al. 2010. Brisbane, Australia. ^11^  Study describing the effect of TDM guided dosing regimens in critically ill patients | Critically ill patients (n=236)**  Patient condition not specified. | Piperacillin-Tazobactam n=116 4.5g q6h  Ampicillin n= 4 2g q6h  Penicillin G n=9 2.4g q4h  Flucloxacillin n=16 2g q4h  n=145 patients receiving penicillins  **Study also included patients receiving non-penicillin antibiotics, n=91** | Total plasma concentrations were determined by HPLC-UV, free antibiotic concentrations were calculated using published protein binding data.  TDM was performed twice a week. For intermittently dosed patients, sampling was initiated at steady-state, after 4 previous doses, within 15 minutes of next dose. For patients under continuous infusion, samples were taken at stead-state, after 4-5 half-lives of antibiotic. | The study hospital uses single-therapy treatments, and only prescribed additional antibiotics in enrolled patients to treat other pathogens that were resistant to the beta-lactam prescribed.  There is no analysis to show how many patients were administered concomitant antibiotics, nor the effect of the concomitant antibiotics on outcome. | Mean serum creatinine concentration at the start of treatment was 111µmol/L +/-91µmol/L. **  ARC (CrCl >150-160 mL/min) was seen in 21/47 patients (44.7%) where 8-hr CrCl was taken.  Of the 21 patients displaying ARC, dose increases were needed in 16 of these patients (76.2%), and dose decreases were needed in 3 patients (14.3%), target attainment was seen in 2 patients (9.5%). **  Renal dysfunction (>180µmol/L serum creatinine) was seen in 10 patients, 9 of which needed dose adjustments. **  CVVHD was being given to 36 patients during initial TDM sampling, 25 (69%) needing dose adjustment. **  Surgical drains were seen in 60 patients, of 52 not needing CVVHD, 41 (79%) needed a dose adjustment. ** | Commonly isolated pathogens: MSSA (n=39), *Klebsiella* spp. (n=16), *Escherichia coli* (n=14). **  MIC values were obtained from local antibiogram data, or where not available EUCAST data. | A pharmacological target of 100% ƒT>4-5xMIC was used.  If TDM result was below the target then dosing frequency was increased by 25% - 50%. If TDM result was within 20% of the target then administration infusion was extended. When intermittent dosing was at maximum dose according to product then continuous dosing was initiated.  If TDM result was >100% ƒT>10xMIC then dose was decreased by 50% or dosing frequency was decreased by 25% - 50%. | In piperacillin patients n=116: Dose maintained: 27 (23%), Dose increased: 57 (49%), Dose decreased: 32 (28%).  In ampicillin patients n=4: Dose maintained: 0 (0%), Dose increased: 1 (25%), Dose decreased: 3 (75%).  In penicillin G patients n=9: Dose maintained: 3 (33%), Dose increased: 3 (33%), Dose decreased: 3 (33%).    In flucloxacillin patients n=16: Dose maintained: 1 (6%), Dose increased: 15 (94%), Dose decreased: 0 (0%)  Of the 51 patients where a second sample was taken for TDM analysis 22 (43.1%) achieved the target concentration. ** | Not specified | A positive clinical resolution was seen in 206/236 of antibiotic courses.**  There was no significant association between negative clinical outcome and subtherapeutic TDM levels (P=0.34), or increased serum creatinine concentration (P=0.34). The only factor significantly associated to negative clinical outcome was APACHE II score (P=0.05). **  The mortality rate for patients with initial subtherapeutic TDM levels was 3.3%, compared to 8.2% who had an initial TDM within the target range, and 9.3% who had an initial TDM above the target range. Factors predictive of mortality were APACHE II score (P<0.01) and increased serum creatinine concentration (P=0.05). ** | Not specified |
| Richter et al. 2019. Germany. ^12^  A retrospective analysis of TDM guided continuous infusion of piperacillin-tazobactam in critically ill patients | Critically ill patients (n=484)  All patients were included from an ICU unit, patients were not scored on condition, using APACHE II score for example. | Piperacillin-Tazobactam, n=484  Continuous infusion 12g/day | Total serum piperacillin concentrations were determined by HPLC.  Samples were taken at least 12 hours and 4 half-lives after piperacillin initiation. | Septic patients were concomitantly administered ciprofloxacin, there was no analysis to determine the effect that the concomitantly administered ciprofloxacin had on outcome. | Mean CrCl: 46.3 mL/min.  RRT was being given to 79/484 of patients (16.3%) of those 71/79 were given CVVHD.  ARC (CrCl ≥130 mg/min) was seen in 41/484 patients (8.5%).  Increased CrCl was associated with variable piperacillin concentrations as well high CrCl being associated with inadequately low piperacillin concentrations <16 mg/L [P<0.0005, OR 1.022 95% CI 1.011-1.034] and 16-32 mg/L [P<0.0005, OR 1.017 95% CI 1.013-1.022].  RRT associated with lower piperacillin clearance (not statistically significant).  CrCl significantly lower in patients who died compared to survivors, median CrCl of those who died: 31.8 mL/min compared to median CrCl of survivors: 48.5 mL/min [P<0.0001]. | Pathogens responsible for infections not specified.  The epidemiological cut-off of piperacillin to *Pseudomonas aeruginosa* (16mg/L) was used as the MIC value. | A pharmacological target of ƒT>2-4xMIC was used, 33-64 mg/L.  TDM results were interpreted and dose adjustments were made by clinical pharmacists. | Target attainment (33-64 mg/L) was initially seen in 166/484 (34.3%) of patients. 269/484 (55.6%) had levels >64 mg/L, of which 146/484 (30.2%) had potentially toxic levels >100 mg/L. 49/484 (10.1%) had levels <16 mg/L.  Following dose adjustments 449 TDM measurements took place, target attainment (33-64 mg/L) was seen in 280/449 patients (62%). 98/449 (21.8%) had levels >64 mg/L, of which 20/449 (4.5%) had potentially toxic levels >100mg/L. 71/449 (15.8%) had levels <33 mg/L. | Not specified | There were 93/484 (19.2%) deaths recorded in ICU, and 112/484 (23.2%) in hospital over the whole study.  Mortality was significantly associated with increased piperacillin concentrations.  Where the target was met (33-64 mg/L) ICU mortality was 12.0% and hospital mortality was 13.9%. In subtherapeutic patients (16-32 mg/L) ICU mortality was 14.6%, [P<0.009], hospital mortality was 20.8% [P<0.005].  In patients above the target (>64 mg/L) ICU mortality was 16.3% [P<0.009, compared to within target patients], hospital mortality was 29.4% [P<0.005, compared to within target patients].  In patients where piperacillin concentrations extremely exceeded the target (>100mg/L) ICU mortality was 31.9% and hospital mortality was 37.6%, significantly more than those moderately above the target (65-99mg/L) [P<0.005 and P<0.001 for ICU and hospital mortality, respectively]. | Not specified |
| Machado et al. 2017. São Paulo, Brazil. ^13^  A retrospective before-and-after study looking at clinical differences between patients before and after TDM of beta-lactams was introduced. | Burns patients (n=140**)  Conventional treatment group median APACHE II score: 18 (range: 8-36)  Monitored treatment group median APACHE II score: 20 (5-26) [P=0.63] | Piperacillin (n=16)  Dosing not specified.  **Study also included non-penicillin antibiotics ** | Plasma antibiotic concentrations were determined by HPLC.  After a minimal 5 half-lives, six serial samples were taken during the course of the dosing interval.  Not specified whether or not TDM was carried out more than on one occasion. | Not specified | Not specified | TDM intervention group (n=77) : *Acinetobacter baumannii* (n=12, 39%), *Staphylococcus aureus* (n=5, 16%), *Pseudomonas aeruginosa* (n=2, 6%), *Enterobacteriaceae* (n=8, 26%), other (n=4, 13%).  Conventional treatment group (n=63): *Acinetobacter baumannii* (n=7, 25%), *Staphylococcus aureus* (n=9, 33%), *Pseudomonas aeruginosa* (n=4, 14%), *Enterobacteriaceae* (n=5, 18%), other (n=2, 10%)  A number of hypothetical MIC concentrations, based on clinical breakpoints, were used to determine target attainment for different susceptibilities | A pharmacological target of 100% ƒT>MIC was used for piperacillin.  Dose adjustment protocol not specified. | No target attainment data for the conventionally treated group as TDM was not carried out.  Of piperacillin patients where TDM was performed, 60% would have achieved target attainment of 100% fT>MIC with a hypothetical MIC of 8 mg/L, 80% achieved the target with a hypothetical MIC of 4 mg/L, 93% at 2 mg/L, and 100% at ≤1 mg/L.  No data reported on target attainment before and after dose adjustment, of those where dosing required adjustment. | Not specified | There were no significant differences in clinical outcome between the conventionally treated group and the TDM intervention group. **  Conventional treatment group: hospital mortality 23 (36%); TDM group: hospital mortality 30 (39%), P=0.83.  Conventional treatment group: 14-day mortality 9 (14%); TDM group 14-day mortality 12 (16%), P=0.99.**  Conventional treatment group: clinical improvement in 29 (52%); TDM group, clinical improvement in 43 (60%), P=0.37. ** | Not specified |
| Schoenenberger-Arnaiz et al. 2019. Spain. ^14^  Determining the usefulness of TDM-guided continuous infusions of piperacillin and meropenem | Critically ill patients (n=92)  APACHE II score: 18.1 (+/- 7.5) ** | Piperacillin-Tazobactam, n=92  Continuous infusion: 16g/2g or 24g/3g/24h  **Study also included non-penicillin antibiotics, n=32 | Total piperacillin serum concentrations were determined by HPLC-UV, free antibiotic concentrations were calculated using published protein binding data.  TDM measurements were taken twice weekly. 49/93 (52.6%) only one TDM measurement was available. 41/93 (44.1%) had two TDM measurements and 3/93 (3.3%) had three. ** | Not specified | Mean piperacillin clearance: 2.5 +/- 2.0 ml/kg/min.  There was a significant difference in piperacillin clearance in patients requiring a dose adjustment and those requiring CRRT, compared to those not requiring a dose adjustment [P<0.0001] | Isolated pathogens treated with piperacillin-tazobactam: *E*. *coli* (n=13), *P*. *aeruginosa* (n=7), other (n=5).  MICs were determined by broth microdilution, if necessary this was confirmed by an E-test. If a MIC could not be determined then an empiric MIC was set, for piperacillin this was 16mg/L. | 100% ƒT>4xMIC was used as the minimum pharmacological target.  Dose adjustment protocol not specified. Dose adjustments were determined by clinicians. TDM result was available to influence dosing regimens. As well as being influences by TDM results, dose adjustments were also carried out according to existing procedures. | Of the 127 cases treated by piperacillin, 63 cases were considered to not need a dose adjustment and had normal renal function, and had a mean piperacillin concentration of 72.5mg/L +/-46.8mg/L. There were 24 cases requiring a dose adjustment, with no CRRT needed, which had a mean piperacillin concentration of 138.6 mg/L +/-44.5 mg/L. There were 40 cases requiring CRRT, which had a mean piperacillin concentration of 138.4 mg/L +/- 57.9 mg/L. | Not specified | Of patients where the pharmacological target was reached, compared to those where it was not, there was no difference in mortality. **  Of patients receiving dose adjustment, most were dose reductions.    Dose adjusted group all-cause mortality: 25%;  no dose adjustment group all-cause mortality: 10.5% ** | Not specified |
| Jansen et al. 2021. Leiden, The Netherlands. ^15^  TDM-guided benzylpenicillin (BZP) treatment in neonates with GBS sepsis and/or meningitis | Neonates with sepsis and/or meningitis from GBS, (n=8). | BZP, n=8.  Minimal dose: 1 hour intravenous infusion 50,000 IU/kg/d. Switched to continuous infusion in severe infection or when no clinical improvement observed. | Serum BZP and/or CSF samples were determined by HPLC-UV or UPLC-MS/MS.  Bolus dosing: TDM sampling was performed at trough concentrations  Continuous Infusion: TDM sampling was performed 24-48 hours after treatment initiation. | All patients were administered concomitant gentamicin. | Not specified. | Microbiologically confirmed Group B Streptococci (GBS) infections. Confirmed through blood cultures or CSF culture.  E-test assays were used to determine the MIC to BZP. Bacterial inhibition through the disk diffusion method, as per EUCAST guidelines, was used when MICs were not available. | A target range of 10-20 mg/L was used.  When serum concentrations measured outside the target range doses were adjusted. | Most measured BZP concentrations were not optimal, 81% (n=17). 43% (n=9) were sub-therapeutic, 38% (n=8) were supra-therapeutic.  Dose adjustments were required in 10 instances. Adjustments resulted in concentrations trending towards the target range, however adjustments generally improve target attainment, 1/4 patients reached target concentrations after adjustments. | Microbiological resolution of infection in 3/8 patients. | Clinical success was observed in 3/8. Treatment failure was seen in 5/8, with 2/5 treatment failures resulting in death.  Clinical success was not associated with those who received dose adjustments from TDM results, 4/8. | Not specified. |
| Gomez-Junyent et al. 2020. Barcelona, Spain. ^16^  TDM of continuously infused β-lactams to treat osteoarticular infections (OI) caused by fluoroquinolone-resistant *Pseudomonas aeruginosa* | Patients with OI infection caused by *P. aeruginosa* (n=52)** | Piperacillin-tazobactam, n=9 TDM samples  Median continuous infusion dose: 12g/day (IQR: 10-12 g/day)  ** Study also included non-penicillin antibiotics, n=183 | Total piperacillin plasma concentrations were determined by UPLC-MS/MS. Measurements were performed twice weekly.  The first TDM sample was taken at least 24 hours after the CI was initiated. Samples were then taken at clinician’s discretion. | Protocol recommended initial treatment of empiric β-lactam with glycopeptides.  Fluoroquinolone-resistant infections were treated with concomitant colistin, susceptible infections were treated with concomitant ciprofloxacin.  Combination therapy in 39 patients (75%).** | Chronic kidney disease was seen in 8 patients (15.4%).** | Microbiologically confirmed *P. aeruginosa* OI. Superinfections were also included where *P. aeruginosa* was involved.  Piperacillin median MIC: 2 mg/L (IQR: 1-4 mg/L) | 100% T>MIC was targeted as well as a ƒC_ss_ (free steady state concentration) of 3-4x MIC, and up to ƒC_ss_ 10x MIC.  When concentrations were 10-15x MIC, >100 mg/L, or when toxicity was observed, doses were reduced. | Median estimated free piperacillin concentrations: 13.2 mg/L (IQR: 11.5-17.5 mg/L)  Median estimated piperacillin:MIC ratio: 2.3 (IQR: 2.0-3.3). | Not specified. | Clinical failure in patients with fluoroquinolone-susceptible strains (where piperacillin was administered for some infections) 5/33 (16.7%), adverse effects 6/33 (18.2%), median duration of therapy 55 days (IQR: 42-59 days). **  Clinical outcomes were not associated to target attainment | Not specified. |
| Chiriac et al. 2021. Germany. ^17^  Software assisted TDM-guided dose adjustments to piperacillin treatment in critically ill patients. | Critically ill patients with sepsis, septic shock, or severe sepsis being treated with piperacillin-tazobactam by continuous infusion (n=179)  Median SOFA score: 6  Median SAPS score: 37  Median APACHE II score: 22 | Piperacillin-Tazobactam, n=179.  Loading dose: 2g by 15 min infusion. Immediate continuous infusion followed with calculated empiric dose. | Total piperacillin concentration were determined by a validated HPLC method.  The first TDM sample was measured 24-48 hours after piperacillin initiation. Subsequent samples were advised by clinical pharmacists. | Not specified. | Median baseline CrCL: 47 mL/min.  33 patients (19%) received RRT, 18 received CRRT, and 15 intermittent haemodialysis. | Most commonly detected pathogens: *E coli*, *Proteus* spp, *Klebsiella* spp n=41 (23%); MSSA, n=14 (8%); Coagulase-negative staphylocci, n=14 (8%).  No detection n=58 (32%).  The EUCAST *P. aeruginosa* breakpoint was used as the MIC value. | A target concentration range of 2-4x the MIC (32-64 mg/L) was set.  If patient piperacillin concentrations measure <16 mg/L a mandatory dose increase was set, if concentrations were 16-32 mg/L it was strongly advised to increase the dose. No action was taken if doses were between 16-32 mg/L. Between 64-96 mg/L a there was a strong recommendation to reduce doses. It was mandatory to reduce doses when concentrations were >96 mg/L. | Within the first 48 hours 40.2% of patients (n=72) had a piperacillin concentration within the target range, after TDM-guided dose adjustments (>48 hours) 65.4% (n=102) were within the target range. TDM also reduced piperacillin concentrations above 96 mg/L from 12.3% (n=22) to 4.5% (n=7). | Not specified. | Hospital mortality was significantly higher in patients with piperacillin concentrations >96 mg/L and 64-96 mg/L compared to the target range (P=0.001, P=0.007, respectively). There was no significant difference in mortality in patients with piperacillin concentrations between 16-32 mL and the target range (P=0.841). | Not specified. |

Abbreviations: APACHE II, Acute Physiology and Chronic Health Evaluation; ARC, Augmented Renal Clearance; BZP, benzylpenicillin; CrCl, Creatinine Clearance; CSF, cerebral spinal fluid; CVVHDF, Continuous Veno-Venous Hemodiafiltration; ECMO, Extracorporeal Membrane Oxygenation; EUCAST, European Committee on Antimicrobial Susceptibility Testing; ƒT>MIC, time unbound antibiotic concentration is above MIC; GBS, Group B Streptococci; GNB, Gram-Negative Bacteria; (P)ICU, (Paediatric) Intensive Care Unit; IQR, Interquartile Range; (H/UP)LC-MSMS/UV, High/Ultra-Performance Liquid Chromatography coupled tandem mass spectrometry/Ultraviolet; MIC, Minimum Inhibitory Concentration; MSSA, Methicillin Susceptible *Staphylococcus aureus*; OI, Osteoarticular infection; PK/PD, Pharmacokinetic/Pharmacodynamic; (C)RRT, (Continuous) Renal Replacement Therapy; SAPS, Simplified Acute Physiology Score; SOFA, Sequential Organ Failure Assessment; TBSA, Total Body Surface Area; TDM, Therapeutic Drug Monitoring; VAP, Ventilator Associated Pneumonia.

Table S3 – Study characteristics of case studies and case series.

| **Reference** | **Patient Condition** | **Penicillin** | **TDM measurement** | **Concomitant Antibiotics** | **Renal Function** | **Pathogen and MIC** | **Pharmacological Target** | **Pharmacological Target Attainment** | **Microbiological Resolution** | **Clinical Resolution** | **Emergence of Antimicrobial Resistance** |
| --- | --- | --- | --- | --- | --- | --- | --- | --- | --- | --- | --- |
| Lonsdale et al. 2020. Brisbane, Australia. ^18^ | Aspiration Pneumonia | Amoxicillin-Clavulante  1.2g q8h | Unbound serum fraction measured by UPLC-MS/MS | No concomitant antibiotics | Serum creatinine concentration before treatment was 68µmol/L. | *Staphylococcus aureus*, and *Klebsiella pneumoniae* (susceptible to co-amoxiclav but no MIC determined) | 40% ƒT>MIC  EUCAST clinical breakpoint for Gram-negative organisms for co-amoxiclav used (8mg/L) | Target not attained, <40% ƒT>MIC achieved. Unbound serum amoxicillin after dose: 1hr - 28.0mg/L; 2hr - 11.0mg/L; 4hr - 5.2mg/L; 6hr - NA; 8hr - 1.0mg/L | Not specified | Yes – assumed actual MIC of infective pathogen <8mg/L | Not specified |
| Parsonson et al. 2020. Brisbane, Australia. ^19^ | Patient 1 – Prosthetic hip infection and osteomyelitis  Patient 2 – endometritis  Patient 3 – bacteraemia secondary to urinary tract infection | Patient 1 – Benzylpenicillin, subsequently Ampicillin.  Ampicillin commenced at 2g q6h, changed to infusion of 5g q12h, increased to 6g q12h.  Patient 2 – Piperacillin/Tazobactam, subsequently Ampicillin. Ampicillin started at  2g q6h, changed to infusion 4g q12h  Patient 3 – Ampicillin.  2g q6h, changed to continuous infusion (CI) 6g q12h. | Patient 1, 2, and 3  Plasma concentration measured by HPLC, unspecified fraction (unbound or total). | Patient 1 – Prophylactic cefazolin and vancomycin. Additional peri-operative vancomycin.  Patient 2 – Patient was treated with cephazolin, metronidazole, and gentamicin. After remaining febrile antibiotics were escalated to piperacillin/tazobactam and lincomycin. After susceptibility testing, antibiotics were changed to ampicillin and oral metronidazole.  Patient 3 – Gentamicin, which was ceased once susceptibilities were available. | Patient 1 – Creatinine Clearance (CrCl) >90ml/min  Patient 2 – CrCl = 190ml/min  Patient 3 – Associated acute kidney impairment CrCl = 40ml/min | MICs determined by Vitek2 microbroth dilution or Etest  Patient 1 – *Enterococus faecalis* benzylpenicillin MIC: 4mg/L ampicillin MIC: ≤2mg/L. *Staphylococcus epidermidis* superinfection MIC not specified.  Patient 2 – *Bacteroides fragilis* and *E. faecalis*. Ampicillin MIC: 0.5mg/L  Patient 3 – *E. faecalis*. Ampicillin MIC: ≤2mg/L | 4-5xMIC levels are targeted.  Not specified whether total plasma concentrations or unbound fractions were targeted.  Patient 1 – 8-10mg/L.  Patient 2 – 2-2.5mg/L  Patient 3 – 8-10mg/L | Patient 1 – 4-5xMIC plasma ampicillin achieved.  Median ampicillin level 13mg/L (range: 7.7-19mg/L)  Patient 2 - >10x MIC plasma ampicillin achieved.  Median ampicillin level 13mg/L (range: 13-18mg/L)  Patient 3 - >10x MIC plasma ampicillin. Median ampicillin level 55.5mg/L (range: 40-66mg/L) | Patient 1 – No, the pathogen persisted after benzylpenicillin and ampicillin therapy.  Patient 2 – Not specified.  Patient 3 – Not specified. | Patient 1 – No, not from ampicillin therapy.  Patient 2 – Yes, patient recovered from ampicillin therapy  Patient 3 – Yes, patient recovered from ampicillin therapy | Patient 1 – Enterococcus remained susceptible after 6 weeks of IV ampicillin.  Patient 2 and 3 – not specified |
| Hayashi et al. 2013. Brisbane, Australia. ^20^ | P1: Not treated with penicillin  P2: Bilateral mastoiditis  P3: Staphylococcal endocarditis complicated by brain emboli | P2: Flucloxacillin  2g q4h  P3: Flucloxacillin  2g q4h initially, then moved to 1g q4h | Total plasma concentration measured by HPLC-UV. | P2: Not specified  P3: Rifampicin and sodium fusidate were co-administered. | P2: Plasma Creatinine: 134 µmol/L. Urine output: 100 mL/h.  P3: Renal dysfunction ClCr = 47 ml/min which decreased to 26 ml/min | P2: Methicillin-sensitive *Staphylococcus aureus* (MSSA). Flucloxacillin MIC: 2 mg/L  P3: *Staphylococcus Aureus* (MSSA) flucloxacillin MIC: 2 mg/L | P2: 100% ƒT>MIC  40-50 mg/L  P3: 100% ƒT>MIC  40-50 mg/L. | P2: On day 4 plasma trough levels were 24 mg/L. CSF levels were <5 mg/L. The dosing regimen was altered to a 12g/day continuous infusion. TDM result on day 6 showed flucloxacillin concentrations of 39 mg/L. The dose was then increased to 16g/24h which resulted in plasma concentrations of 51 mg/L.  P3: At 2g q4h elevated flucloxacillin concentrations at 147mg/L.  At 1gq4h (and implementation of CRRT) flucloxacillin concentrations 46mg/L. | P2: Not specified  P3: Not specified | P2: There was signs of clinical improvement and sufficient enough to discharge from ICU.  P3: No – Patient exhibited persistent poor neurological function and discharged with no further planned intervention | P2: Not specified.  P3: Not specified. |
| Al Yazidi et al. 2019. ^21^ | Neonate on ECMO with severe infection | Flucloxacillin  50 mg/kg/4h | Total plasma concentration measured. TDM method not specified. | Linezolid was co-administered, prior to flucloxacillin TDM result, it was then ceased. | Not specified | *Staphylococcus Aureus* (MSSA) flucloxacillin Breakpoint MIC: 2 mg/L | 40% ƒT>MIC  Breakpoint MIC value used | Total plasma flucloxacillin: 22.2mg/L  Estimated unbound fraction: 5.6mg/L | Yes, blood cultures confirmed no further infection | Yes, patient discharged after rehabilitation unrelated to infection | Not specified |
| Abdul-Aziz et al. 2014. Brisbane, Australia. ^22^ | Cellulitis | Flucloxacillin  Initial 2g q4h, changed to continuous infusion 16g/24h, then increased to 20g/24h. | Plasma concentration, unspecified fraction (unbound or total), and CSF were measured by HPLC-UV | No concomitant antibiotics | Day 1 CrCl: 234 mL/min Day 3 CrCl: 227 mL/min Day 6 CrCl: 241 mL/min Day 7 CrCl: 262 mL/min Day 15 CrCl: 153 mL/min | *Staphylococcus Aureus* (MSSA) flucloxacillin MIC: 1 mg/L | 100% ƒT>MIC  Plasma and CSF trough concentrations of 40 mg/L and 4 mg/L, respectively, were targeted. Assumed 10% flucloxacillin penetration. | At 2g q4h: median plasma flucloxacillin trough concentrations: 0.3 mg/L (range: 0.2-0.4 mg/L). CSF concentration : <0.1 mg/L.  At 16g/24h CI plasma concentration: 1.6 mg/L, CSF: <0.1 mg/L.  At 20g/24h CI median plasma concentration: 2.7 mg/L (range: 1.1 – 5.7), CSF: 0.1 mg/L | Not specified | Yes, clinical improvement was observed and later discharged. | Not specified |
| Seddon et al. 2020. Florida, USA. ^23^ | Bioprosthetic and Native Valve Infective Endocarditis | Oxacillin  2g q4h | Total serum concentrations were measured by LC-MS | No concomitant antibiotics | Normal renal function, CrCl >60mL/min | *Staphylococcus Aureus* (MSSA) Oxacillin MIC: 0.5 mg/L | 50% ƒT>MIC | Free oxacillin concentration was calculated from total serum TDM measurement. Pharmacological target was met at 58% ƒT>MIC | Yes, repeat blood cultures confirmed there was no infection | Yes, clinical improvement and discharge from hospital | Not specified |
| Neuner et al. 2012. Cleveland, USA. ^24^ | Radiation-induced encephalomalacia with intracranial cyst | Piperacillin/Tazobactam  3g q4h, then 20g/24h continuous infusion | Total serum and CSF levels were measured by HPLC | Empiric meropenem was initiated, switched to piperacillin/tazobactam, rifampicin and vancomycin. Ciprofloxacin was then added to the regimen. Finally therapy switched to levofloxacin. | Not specified | *Elizabethkingia meningosepicum* Piperacillin/Tazobactam MIC: 8 mg/L | T>MIC | Piperacillin levels, during intermittent dosing were low (values not specified). During continuous infusion serum concentration was 99.4mg/L, 100% T>MIC. CSF levels were 2.3mg/L, 0% T>MIC | No microbiological resolution from penicillin therapy | No clinical improvement from penicillin therapy | Not specified |
| Green et al. 1985. ^25^ | Acute leukemia patient with septic Pseudomonal infection | Piperacillin  Continuous infusion, 18g/24h, reduced to 3g/24h | Serum piperacillin levels were measured, method of TDM not specified. | Empiric ticarcillin and gentamicin, switched to piperacillin and amikacin. | At admission serum creatinine was 0.8mg/dL. Acute renal failure developed and serum creatinine rose to 8.8mg/dL over 5 days. | *Pseudomonas aeruginosa* piperacillin MIC not specified. Mentioned therapeutic range: 64-256mg/L | T>4-5xMIC | Piperacillin levels were measured over 19 days, measured range was 57-175mg/L, median: 131mg/L | Not specified | Clinical improvement following TDM adjusted dosing regimen | Not specified |
| Dumangin et al. 2020. Nancy, France. ^26^ | Urinary tract infection | Temocillin  Continuous infusion of 10mg/kg/day | Total serum and urine concentrations were measured by UPLC-UV | Prophylactic co-trimoxazole, then treated with ceftriaxone, followed by cefixime, then switched to temocillin | After initial stabilisation from end-stage renal disease, serum creatinine levels were between 141-176µmol/L and glomerular filtration rate was 11-17 mL/min/1.73m^2^ | ESBL-producing *Enterobacter cloacae* temocillin MIC: 6mg/L | 100% ƒT>MIC  A measured MIC of 6mg/L was used as the target concentration for unbound temocillin | Free serum concentrations of temocillin were estimated from measured total levels. Total measured serum concentration: 47.0 – 61.8 mg/L; estimated free concentration 7.0 -9.2 mg/L. Urinary concentrations: 21.6-35.5 mg/L. Temocillin concentrations were measured above the target at all times. | No recurrence was observed | Clinical resolution in response to temocillin was seen | Not specified |

Abbreviations: CrCl, Creatinine Clearance; CSF, Cerebral Spinal Fluid; ECMO, Extracorporeal Membrane Oxygenation; EUCAST, European Committee on Antimicrobial Susceptibility Testing; ƒT>MIC, time unbound antibiotic concentration is above the MIC; ICU, Intensive Care Unit; IV, Intravenous; (H/U-P)LC-MS/MS, (High/Ultra-Powered) Liquid Chromatography coupled tandem Mass Spectrometry; MIC, Minimum Inhibitory Concentration; (C)RRT, (Continuous) Renal Replacement Therapy; TDM, Therapeutic Drug Monitoring.

References:

1. Sime FB, Roberts MS, Tiong IS et al. Can therapeutic drug monitoring optimize exposure to piperacillin in febrile neutropenic patients with haematological malignancies? A randomized controlled trial. *Journal of Antimicrobial Chemotherapy* 2015; **70**: 2369-75.

2. Fournier A, Eggimann P, Pantet O et al. Impact of real-time therapeutic drug monitoring on the prescription of antibiotics in burn patients requiring admission to the intensive care unit. *Antimicrobial Agents and Chemotherapy* 2018; **62**.

3. De Waele JJ, Carrette S, Carlier M et al. Therapeutic drug monitoring-based dose optimisation of piperacillin and meropenem: a randomised controlled trial. *Intensive Care Med* 2014; **40**: 380-7.

4. Cies JF, Moore WS, Enache A et al. beta-lactam therapeutic drug management in the PICU. *Critical Care Medicine* 2018; **46**: 272-9.

5. Duszynska W, Taccone FS, Switala M et al. Continuous infusion of piperacillin/tazobactam in ventilator-associated pneumonia: a pilot study on efficacy and costs. *International Journal of Antimicrobial Agents* 2012; **39**: 153-8.

6. Economou CJP, Wong G, McWhinney B et al. Impact of beta-lactam antibiotic therapeutic drug monitoring on dose adjustments in critically ill patients undergoing continuous renal replacement therapy. *International Journal of Antimicrobial Agents* 2017; **49**: 589-94.

7. Besnard T, Carrie C, Petit L et al. Increased dosing regimens of piperacillin-tazobactam are needed to avoid subtherapeutic exposure in critically ill patients with augmented renal clearance. *Critical Care* 2019; **23**.

8. McDonald C, Cotta MO, Little PJ et al. Is high-dose beta-lactam therapy associated with excessive drug toxicity in critically ill patients? *Minerva Anestesiologica* 2016; **82**: 957-65.

9. Wong G, Briscoe S, McWhinney B et al. Therapeutic drug monitoring of beta-lactam antibiotics in the critically ill: direct measurement of unbound drug concentrations to achieve appropriate drug exposures. *Journal of Antimicrobial Chemotherapy* 2018; **73**: 3087-94.

10. Patel BM, Paratz J, See NC et al. Therapeutic drug monitoring of beta-lactam antibiotics in burns patients--a one-year prospective study. *Ther Drug Monit* 2012; **34**: 160-4.

11. Roberts JA, Ulldemolins M, Roberts MS et al. Therapeutic drug monitoring of beta-lactams in critically ill patients: proof of concept. *Int J Antimicrob Agents* 2010; **36**: 332-9.

12. Richter DC, Frey O, Röhr A et al. Therapeutic drug monitoring-guided continuous infusion of piperacillin/tazobactam significantly improves pharmacokinetic target attainment in critically ill patients: a retrospective analysis of four years of clinical experience. *Infection* 2019; **47**: 1001-11.

13. Machado AS, Oliveira MS, Sanches C et al. Clinical Outcome and Antimicrobial Therapeutic Drug Monitoring for the Treatment of Infections in Acute Burn Patients. *Clinical Therapeutics* 2017; **39**: 1649-57.e3.

14. Schoenenberger-Arnaiz JA, Ahmad-Diaz F, Miralbes-Torner M et al. Usefulness of therapeutic drug monitoring of piperacillin and meropenem in routine clinical practice: A prospective cohort study in critically ill patients. *European Journal of Hospital Pharmacy* 2019.

15. Jansen SJ, Lopriore E, Bredius RGM et al. Benzylpenicillin Serum Concentrations in Neonates With Group B Streptococci Sepsis or Meningitis: A Descriptive Cohort Study. *Pediatr Infect Dis J* 2021; **40**: 434-9.

16. Gomez-Junyent J, Rigo-Bonnin R, Benavent E et al. Efficacy and Therapeutic Drug Monitoring of Continuous Beta-Lactam Infusion for Osteoarticular Infections Caused by Fluoroquinolone-Resistant Pseudomonas aeruginosa: A Prospective Cohort Study. *European Journal of Drug Metabolism and Pharmacokinetics* 2020; **45(5)**: 587-99.

17. Chiriac U, Richter DC, Frey OR et al. Personalized Piperacillin Dosing for the Critically Ill: A Retrospective Analysis of Clinical Experience with Dosing Software and Therapeutic Drug Monitoring to Optimize Antimicrobial Dosing. *Antibiotics (Basel)* 2021; **10**: 03.

18. Lonsdale DO, Lipman J, Livermore A et al. Amoxicillin-Clavulanate Dosing in the Intensive Care Unit: The Additive Effect of Renal Replacement Therapy in a Patient with Normal Kidney Function. *Chemotherapy* 2020; **64**: 173-6.

19. Parsonson F, Legg A, Halford M et al. Contemporaneous management of ampicillin infusions in the outpatient setting through the use of therapeutic drug monitoring. *Int J Antimicrob Agents* 2020; **55**: 105975.

20. Hayashi Y, Lipman J, Udy AA et al. beta-Lactam therapeutic drug monitoring in the critically ill: Optimising drug exposure in patients with fluctuating renal function and hypoalbuminaemia. *International Journal of Antimicrobial Agents* 2013; **41**: 162-6.

21. Al Yazidi LS, Badran SA, Sandaradura I et al. Flucloxacillin therapeutic drug monitoring in a neonate on extracorporeal membrane oxygenation. *Journal of Paediatrics & Child Health* 2019; **55**: 246-7.

22. Abdul-Aziz MH, McDonald C, McWhinney B et al. Low flucloxacillin concentrations in a patient with central nervous system infection: the need for plasma and cerebrospinal fluid drug monitoring in the ICU. *Ann Pharmacother* 2014; **48**: 1380-4.

23. Seddon MM, Busey KV, Kutner SB et al. Oxacillin therapeutic drug monitoring in a patient on extracorporeal membrane oxygenation support. *The Journal of antimicrobial chemotherapy* 2020; **17**.

24. Neuner EA, Ahrens CL, Groszek JJ et al. Use of therapeutic drug monitoring to treat Elizabethkingia meningoseptica meningitis and bacteraemia in an adult. *Journal of Antimicrobial Chemotherapy* 2012; **67**: 1558-60.

25. Green L, Dick JD, Goldberger SP et al. Prolonged elimination of piperacillin in a patient with renal and liver failure. *Drug Intelligence & Clinical Pharmacy* 1985; **19**: 427-9.

26. Dumangin G, Brenkman M, Pape E et al. Temocillin dosage adjustment in a preterm infant with severe renal disease: a case report. *Journal of Antimicrobial Chemotherapy* 2020; **20**: 20.
